# Safety and efficacy of bispecific antibodies in hematologic neoplasms: a systematic review

**DOI:** 10.3389/fimmu.2026.1815519

**Published:** 2026-08-11

**Authors:** Khanittha Lommuang, Suthira Taychakhoonavudh

**Affiliations:** Department of Social and Administrative Pharmacy, Faculty of Pharmaceutical Sciences, Chulalongkorn University, Bangkok, Thailand

**Keywords:** bispecific antibodies, efficacy, hematologic neoplasms, safety, systematic review

## Abstract

**Background:**

Hematologic neoplasms remain a growing global burden, with relapse, resistance, and limited treatment options persisting. Bispecific antibodies (BsAbs) offer a novel multi-antigen targeting strategy. This systematic review assesses their efficacy and safety, offering an in-depth appraisal of their therapeutic potential and the unmet medical needs.

**Methods:**

We conducted this systematic review following the PRISMA 2020 guidelines. Articles published up to 20 June 2026 were searched across PubMed, Scopus, Web of Science, and Embase. Efficacy outcomes such as measurable residual disease (MRD), overall response rate (ORR), complete response (CR), overall survival (OS), and progression-free survival (PFS), as well as safety outcomes such as severe adverse events (SAEs), grade ≥3 adverse events (≥3 AEs), hematologic and non-hematologic toxicities were systematically evaluated.

**Results:**

Thirty-six studies evaluating nineteen different BsAbs were included. Blinatumomab (CD3-CD19) remains the most extensively studied bispecific antibody in relapsed or refractory B-cell precursor acute lymphoblastic leukemia (R/R BCP-ALL). CD3-CD20 BsAbs (Glofitamab, Epcoritamab) demonstrated durability in non-Hodgkin lymphoma (NHL). In multiple myeloma (MM), BCMA- and GPRC5D-targeting BsAbs achieved MRD negativity and deep responses including very good partial response (VGPR) and stringent complete response (sCR). BsAbs in acute myeloid leukemia (AML) and Hodgkin/T-cell lymphoma (HL/TCL) remained early-phase with limited efficacy and no approvals to date. Cytokine release syndrome (CRS) and neurotoxicity were the dominant toxicities of CD3-engaging agents (neurologic events >50% in several Blinatumomab studies). Teclistamab (CD3–BCMA) and Talquetamab (CD3–GPRC5D) were associated with frequent CRS, with neurotoxicity also reported. However, hematologic toxicity and infections remain major challenges in multiple myeloma treatment.

**Conclusion:**

BsAbs demonstrate established clinical activity in BCP-ALL, B-cell NHL, and MM but remain under investigation in AML, HL, and T-cell lymphoma. CRS, neurotoxicity, cytopenias, and infections remain key safety considerations. Further studies should optimize treatment strategies and evaluate emerging targets and non-CD3-engaging bispecific antibodies.

The protocol was prospectively registered in PROSPERO (CRD42023467556).

**Systematic Review Registration:**

https://www.crd.york.ac.uk/PROSPERO/, identifier CRD42023467556.

## 1 Introduction

Hematologic neoplasms encompass a broad spectrum of neoplasms affecting the blood, bone marrow, and lymphatic system. These malignancies are generally classified by their tissue of origin: leukemias arise in the blood, lymphomas develop in the lymph nodes, and myelomas originate in the bone marrow (1). Among leukemias, acute myeloid leukemia (AML) is the most prevalent acute leukemia in adults, marked by significant genetic and clonal heterogeneity that contributes to treatment resistance (2). Lymphomas are further divided into Hodgkin (HL) and non-Hodgkin (NHL) subtypes, with NHL representing 90% of all lymphoma cases and originating from lymphocytes at different developmental stages (3). Multiple myeloma (MM), which accounts for about 10% of hematologic neoplasms (4), remains incurable and typically follows an asymptomatic precursor state (5).

Over the last three decades, the global burden of hematologic neoplasms has risen, with increases in both incidence and mortality, particularly in older populations (6, 7). Patients with these neoplasms face greater risks of treatment-related complications and higher mortality rates compared to those with solid tumors (8, 9). The median age at diagnosis for hematologic neoplasms is reported as 70.8 years, an overall 5-year relative survival of 69.2%, attributed to advancements in treatments (10). Despite advancements in current treatments—chemotherapy (11), monoclonal antibodies (mAbs), and chimeric antigen receptor T (CAR-T) cell therapies, many patients experience treatment failure due to relapse, resistance, and the limitations of treatment options (12).

To address these challenges, bispecific antibodies (BsAbs) have emerged as a novel therapeutic approach (13). BsAbs can target two different epitopes, increasing specificity and overcoming drug resistance (14). Traditional mAbs and CAR-T therapies often fail due to downregulation of target antigens (15, 16), such as CD19 loss in B-cell neoplasms or B-cell maturation antigen (BCMA) loss in multiple myeloma (16). BsAbs mitigate this by engaging multiple antigens or immune effectors (for example simultaneously binding a tumor-associated antigen such as CD19, CD20, or BCMA together with an immune-effector trigger such as CD3 on T cells or CD16A on natural killer cells), ensuring sustained tumor cell recognition and destruction (17). Additionally, unlike the CAR-T therapies, which require personalized manufacturing and long processing times (16), BsAbs are off-the-shelf therapies, allowing for rapid treatment initiation and broader patient access (18). Among BsAbs, bispecific T-cell engagers (BiTEs), which link T cells to cancer cells, have shown remarkable promise in treating hematologic neoplasms, leading to several recent FDA approvals (19) such as Blinatumomab (Philadelphia chromosome-negative relapsed or refractory B-cell precursor acute lymphoblastic leukemia, Ph- negative R/R BCP-ALL) (20), Mosunetuzumab (relapsed or refractory follicular lymphoma, R/R FL) (21), and Epcoritamab (diffuse large B-cell lymphoma, DLBCL) (22).

While the clinical development of BsAbs has expanded rapidly (19). To date, there has been no comprehensive systematic review evaluating the safety and efficacy of BsAbs across all hematologic neoplasms. Therefore, this review systematically evaluates clinical outcomes of BsAbs, providing an in-depth appraisal of their unmet clinical needs and therapeutic potential.

## 2 Materials and methods

### 2.1 Protocol and registration

This systematic review was conducted in accordance with the PRISMA 2020 guidelines (23), with the corresponding checklist provided in **Supplementary Material 1**. The protocol was prospectively registered in PROSPERO (CRD42023467556).

### 2.2 Search strategy and eligibility criteria

We searched the following electronic bibliographic databases: PubMed, Scopus, Web of Science, and EMBASE. Additionally, we searched ClinicalTrials.gov (http://clinicaltrials.gov/). Search terms and eligibility criteria were developed according to the PICO strategy by KL under the supervision of ST, outlined in **Supplementary Materials 2**, **3**. The population comprised adults with hematologic neoplasms (leukemia, lymphoma, and multiple myeloma). The intervention included CD3-engaging and non-CD3-engaging BsAbs, with no formal comparator required. Outcomes included disease-specific efficacy endpoints (MRD, VGPR, ORR, CR, sCR, PFS, and OS) and safety outcomes (SAEs, grade ≥3 AEs, treatment discontinuation, treatment-related mortality, CRS, neurotoxicity/immune effector cell-associated neurotoxicity syndrome (ICANS), cytopenias, infections, and hypogammaglobulinemia), where reported. Backward and forward citation tracking was performed to identify additional studies. An updated search was conducted one month prior to manuscript submission to include the latest eligible studies. We searched for published articles from database inception up to 20 June 2026. Results were imported into EndNote 2.0 for de-duplication, with further screening during the title and abstract review. No language or publication date filters were applied to ensure comprehensive coverage of BsAb studies in hematologic neoplasms.

### 2.3 Study selection and data extraction

Two researchers (KL and ST) independently screened all retrieved article titles and abstracts according to inclusion and exclusion criteria. Relevant and potentially relevant full texts were then reviewed independently to confirm eligibility, with any discrepancies resolved through discussion or consultation with a third reviewer. An adapted PRISMA flowchart was created to outline the selection process.

For data extraction, a standardized, pre-piloted form in Microsoft Excel was used to collect key study characteristics, participant details, interventions, and outcomes. Extracted information included study author, publication year, trial phase, study design, funding sources, participant demographics, Bispecific Antibody (BsAb) type and targets, dosage, schedule, adverse event occurrences (CRS, grade ≥3 hematologic toxicities, neurotoxicity, infections), response outcomes (MRD, VGPR, ORR, CR, sCR), and survival metrics (PFS, OS).

### 2.4 Quality assessment

The two reviewers (KL and ST) independently assessed all articles by using the Risk Of Bias In Non-randomized Studies – of Interventions (ROBINS-I) assessment tool (24) for non-randomized phase I-II studies and Cochrane Risk of Bias 2 tool (RoB 2) (25) for phase III randomized trials. ROBINS-I V2 was adapted for single-arm dose-escalation studies by focusing on domain-level bias relevant to safety and dose-finding signals, with emphasis on confounding, participant selection, missing data, selective reporting. Judgments were made separately for each key outcome. In case of any disagreement between the reviewers, it was resolved by consultation with a third author or discussion between the reviewers.

### 2.5 Data synthesis

This study evaluated efficacy and safety from phase I-III trials, focusing on efficacy outcomes (MRD, VGPR, ORR, CR, sCR, PFS, and OS) alongside safety data severe adverse events (SAEs), grade ≥ 3 AEs, hematologic and non-hematologic toxicities. Due to study heterogeneity and limited data, planned meta-analysis was unfeasible, so results were summarized qualitatively.

### 2.6 Patient and public involvement

Patients or the public were not involved in the design, or conduct, or reporting, or dissemination plans of this research.

### 2.7 Research ethics approval

This study does not involve human participants or animals. Therefore, research ethics approval is not applicable to this study.

## 3 Results

### 3.1 Study selection

**Figure 1:**
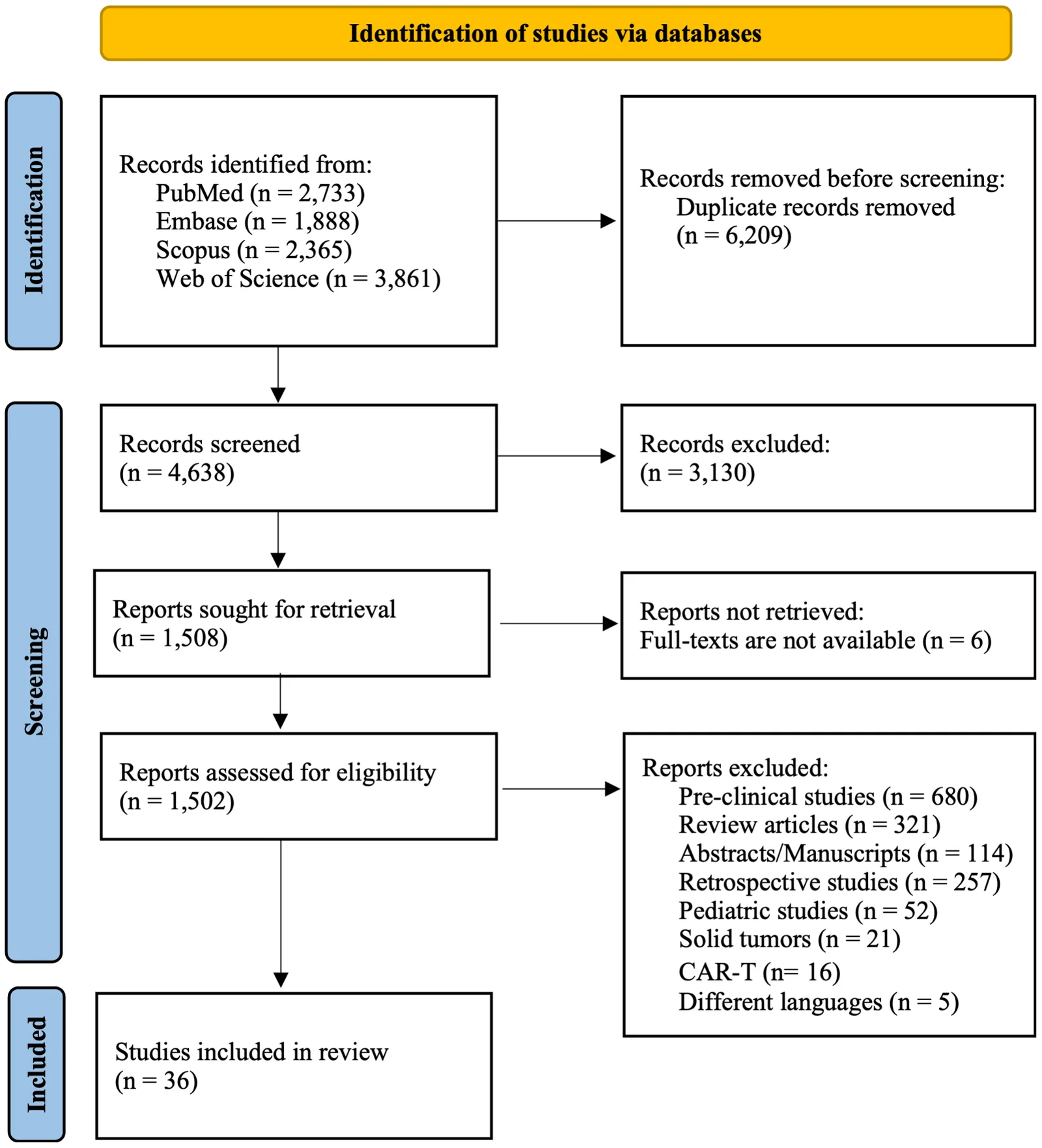
The PRISMA flow diagram (23).

During the initial screening of databases, a total of 10,847 references were identified through PubMed (n = 2,733), Embase (n = 1,888), Scopus (n = 2,365*)*, and Web of Science (n = 3,861). Following the removal of 6,209 duplicate references, 4,638 publications underwent title/abstract screening of which 3,130 records were excluded. Six references could not be retrieved in full text. Subsequently, 1,502 publications were evaluated by full-texts. Due to various reasons, 1,466 publications were excluded. Finally, 36 potential articles were included for qualitative and quantitative data synthesis. The PRISMA flow diagram of study selection was depicted in **Figure 1**.

### 3.2 Study characteristics

The summarized details of the included publications are presented in **Table 1**. All trials were published within the timeframe spanning from 2014 to 2026.

**Table 1:** Study characteristics.

No	First author, year	Study design	Study phase	BsAb name (BsAb code)	BsAb target	Cancer type	Cancer stage	No. of prior lines of therapy	No. of patients	Median age	% Male	Ref.
Leukemia												
1	Topp MS., 2014	Open-label, non-randomized, single-arm study	Phase II	Blinatumomab (AMG103, MT103)	CD3-CD19	BCP-ALL	R/R	NR	36	32	61	(41)
2	Topp MS., 2015	Open-label, non-randomized, single-arm study	Phase II	Blinatumomab (AMG103, MT103)	CD3-CD19	Ph-ve BCP-ALL	R/R	NR	189	39	63	(42)
3	Kantarjian H.,2017	Open-label, randomized study	Phase III	Blinatumomab (AMG103, MT103)	CD3-CD19	Ph-ve BCP-ALL	R/R	NR	271	41	60	(27)
4	Gökbuget N., 2018	Open-label, non-randomized, single-arm study	Phase II	Blinatumomab (AMG103, MT103)	CD3-CD19	BCP-ALL	R/R	NR	116	45	59	(44)
5	Martinelli G., 2021	Open-label, non-randomized, single-arm study	Phase II	Blinatumomab (AMG103, MT103)	CD3-CD19	Ph+ ALL	R/R	NR	45	55	NR	(43)
6	Jabbour E., 2025	Open-label, non-randomized, single-arm study	Phase I/II	Blinatumomab (SQ) (AMG103, MT103)	CD3-CD19	BCP-ALL	R/R	NR	88	46	61	(28)
7	Uckun FM., 2021	Open-label, non-randomized, dose-escalation, and dose-expansion study	Phase IB	APVO436	CD3-CD123	AML	R/R	NR	46	69	52	(29)
8	Uy GL., 2021	Open-label, non-randomized, dose-escalation study	Phase I/II	Flotetuzumab (MGD006)	CD3-CD123	AML	R/R	4 (1–9)	88	64	35	(30)
9	Boyiadzis M., 2023	Open-label, non-randomized, dose-escalation, and dose-expansion study	Phase I	JNJ-63709178	CD3-CD123	AML	R/R	3 (1–10)	62	67	50	(31)
10	Ravandi F., 2024	Open-label, non-randomized, dose-escalation study	Phase IA	AMG 330	CD3-CD33	AML	R/R	NR	77	59	56	(32)
11	Hutchings M., 2025	Open-label, non-randomized, dose-escalation study	Phase I	RO7283420	CD3-HLA-A2-WT1	AML	R/R	2 (1–5)	62	66	55	(33)
Lymphoma												
12	Rothe A., 2015	Open-label, non-randomized, dose-escalation study	Phase I	AFM13	CD16A-CD30	HL	R/R	6 (3–11)	28	38.50	64	(64)
13	Goebeler ME., 2016	Open-label, non-randomized, single-arm study	Phase I	Blinatumomab (AMG103, MT103)	CD3-CD19	NHL	R/R	3 (1–10)	76	65	75	(65)
14	Hutchings M., 2021	Open-label, non-randomized, dose-escalation, and dose-expansion study	Phase I	Glofitamab (RO7082859)	CD3-CD20	B-NHL	R/R	3 (1–13)	171	64	58	(59)
15	Hutchings M., 2021	Open-label, non-randomized, dose-escalation, and dose-expansion study	Phase I/II	Epcoritamab (GEN3013)	CD3-CD20	B-NHL	R/R	3 (2-4.50)	68	68	66	(60)
16	Bannerji R., 2022	Open-label, non-randomized, dose-escalation, and dose-expansion study	Phase I	Odronextamab (REGN1979)	CD3-CD20	B-NHL	R/R	3 (2–5)	145	67	70	(61)
17	Wang L., 2026	Open-label, non-randomized, dose-escalation study	Phase IA	PSB202	CD20-CD37	B-NHL	R/R	3 (2–7)	15	68	60	(34)
18	Thieblemont C., 2022	Open-label, non-randomized, dose-escalation, and dose-expansion study	Phase I/II	Epcoritamab (GEN3013)	CD3-CD20	Large B- NHL	R/R	3 (2–11)	157	64	60	(45)
19	Viardot A., 2016	Open-label, non-randomized, single-arm study	Phase II	Blinatumomab (AMG103, MT103)	CD3-CD19	DLBCL	R/R	3 (1–7)	25	66	56	(62)
20	Katz DA., 2022	Open-label, non-randomized, single-arm study	Phase II	Blinatumomab (AMG103, MT103)	CD3-CD19	DLBCL	Newly diagnosed	NR	28	64	36	(26)
21	Abramson JS., 2024	Open-label, randomized study	Phase III	Glofitamab (RO7082859)	CD3-CD20	DLBCL	R/R	1 (1–2)	274	68	58	(46)
22	Kim WS., 2025	Open-label, non-randomized, single-arm study	Phase II	Odronextamab (REGN1979)	CD3-CD20	DLBCL	R/R	2 (2–8)	127	67	60	(47)
23	Budde LE., 2022	Open-label, non-randomized, single-arm study	Phase II	Mosunetuzumab (BTCT4465A)	CD3-CD20	FL	R/R	3 (2–4)	90	60	61	(48)
24	Linton KM., 2024	Open-label, non-randomized, single-arm study	Phase II	Epcoritamab (GEN3013)	CD3-CD20	FL	R/R	3 (2–4)	128	65	62	(49)
25	Falchi L., 2026	Open-label, randomized study	Phase III	Epcoritamab (GEN3013)	CD3-CD20	FL	R/R	1 (1–2)	243	243	60	(50)
26	Kim WS., 2025	Open-label, non-randomized, single-arm study	Phase II	Acimtamig (AFM13)	CD16A-CD30	PTCL	R/R	2 (1–7)	108	108	63	(63)
Multiple myeloma												
27	Topp MS., 2020	Open-label, non-randomized, single-arm study	Phase I	Pacanalotamab (BI 836909, AMG 420)	CD3-BCMA	MM	R/R	5 (2–14)	42	65	64	(51)
28.1	Chari A., 2022	Open-label, non-randomized, dose-escalation, and dose-expansion study	Phase I	Talquetamab (SQ) (JNJ-64407564)	CD3-GPRC5D	MM	R/R	6 (2–17)	130	64	58	(58)
28.2	Chari A., 2022	Open-label, non-randomized, dose-escalation, and dose-expansion study	Phase I	Talquetamab (IV) (JNJ-64407564)	CD3-GPRC5D	MM	R/R	6 (3–20)	102	65	56	(58)
29	Moreau P., 2022	Open-label, non-randomized, single-arm study	Phase I/II	Teclistamab (JNJ-64007957)	CD3-BCMA	MM	R/R	5 (2–14)	165	64	58	(52)
30	Raab MS., 2023	Open-label, non-randomized, dose-escalation, and dose-expansion study	Phase I	WVT078	CD3-BCMA	MM	R/R	5 (2–13)	33	64	58	(53)
31	Plesner T., 2023	Open-label, non-randomized, dose-escalation study	Phase I	RO7297089	CD16a-BCMA	MM	R/R	7 (2–12)	27	62	70	(40)
32	Lesokhin AM., 2023	Open-label, non-randomized, single-arm study	Phase II	Elranatamab (PF-06863135)	CD3-BCMA	MM	R/R	5 (2–22)	123	68	55	(54)
33	Bumma N., 2024	Open-label, non-randomized, dose-escalation study	Phase I/II	Linvoseltamab (REGN5458)	CD3-BCMA	MM	R/R	5 (2–16)	117	70	55	(55)
34	Rodriguez C., 2025	Open-label, non-randomized, dose-expansion study	Phase IB	Pacanalotamab (AMG 420)	CD3-BCMA	MM	R/R	6 (3–13)	23	64	61	(56)
35	Chari A., 2025	Open-label, non-randomized, dose-escalation study	Phase I/II	Talquetamab (SQ) (JNJ-64407564)	CD3-GPRC5D	MM	R/R	4.5 (4–6)	154	67	58	(57)
36	Touzeau C., 2026	Open-label, randomized study	Phase III	Teclistamab (JNJ-64007957)	CD3-BCMA	MM	R/R	2 (1–3)	296	70	51	(38)

BsAb, Bispecific antibody; CD, Cluster of Differentiation; R/R, Relapsed/Refractory; BCP-ALL, B-cell precursor acute lymphoblastic leukemia; Ph-ve, Philadelphia-chromosome negative; Ph+, Philadelphia chromosome-positive; ALL, Acute lymphoblastic leukemia; AML, Acute myeloid leukemia; NHL, Non-Hodgkin lymphoma; B-NHL, B-cell non-Hodgkin lymphoma; HL, Hodgkin lymphoma; DLBCL, Diffuse large B-cell lymphoma; FL, Follicular lymphoma; PTCL, Peripheral T-cell lymphoma; MM, Multiple myeloma; BCMA, B-cell maturation antigen; GPRC5D, G protein-coupled receptor, class C group 5 member D; HLA-A2, WT1, Human leukocyte antigen-A2–restricted Wilms tumor 1; SQ, Subcutaneous; IV, Intravenous; NR, Not reported.

The trials included patients with three predominant tumor types: leukemia, lymphoma, and multiple myeloma, with lymphoma being the most prevalent. The median age of the patients in the study varied between 32 and 70 years, with majority of them being male. Additionally, only one publication involved newly-diagnosed patients (26), while the remaining studies focused on individuals with relapsed/refractory hematologic neoplasms. The median prior line of therapy ranged from one to seven.

Nineteen bispecific antibodies (BsAbs) were included in the study and administered via intravenous infusion or subcutaneous injection.

In leukemia, CD3–CD19 BsAbs, particularly intravenous infusion Blinatumomab, have demonstrated the most clinical maturity, with four completed phase II trials, one phase III (27), and regulatory approvals for R/R BCP-ALL in 2014 and 2024. A subcutaneous formulation of Blinatumomab was recently evaluated in a phase I/II trial (28). BsAbs targeting CD3–CD123 in AML, including APVO436 (29), Flotetuzumab (30), and JNJ-63709178 (31), remained in early-phase development, and none has received regulatory approval to date. Two additional BsAbs, CD3–CD33 (AMG 330) (32) and CD3–HLA-A2–WT1 (RO7283420) (33), were evaluated in phase I clinical trials; AMG 330 was terminated, whereas RO7283420 recently completed phase I evaluation in 2023.

The clinical development landscape of BsAbs in lymphoma has progressed substantially across all phases, with multiple candidates having completed phase I and II trials and several phase III studies currently ongoing. CD3–CD20 BsAbs have become the predominant class of BsAbs for B-cell lymphomas, building on earlier clinical experience with CD3–CD19 BsAbs. Beyond CD3–CD20 and CD3–CD19 BsAbs, a CD20–CD37 BsAb has more recently been evaluated in a phase Ia clinical study (34). CD16A–CD30 BsAbs were first evaluated in Hodgkin’s lymphoma (HL), with limited subsequent clinical development until more recent studies extended their evaluation to peripheral T-cell lymphoma (PTCL). Among CD3–CD20 BsAbs, Epcoritamab received FDA approval for relapsed/refractory follicular lymphoma in combination with lenalidomide and rituximab in 2025 (35), whereas Mosunetuzumab expanded from its previously approved intravenous formulation to include a subcutaneous formulation in the same year (36).

In multiple myeloma, BsAbs directed against BCMA and GPRC5D have progressed rapidly from early-phase studies to phase III evaluation and regulatory approval. Teclistamab became the first BCMA-targeting BsAb to receive FDA approval in 2022, followed by the approval of the GPRC5D-targeting BsAb Talquetamab and the BCMA-targeting BsAb Elranatamab in 2023. FDA subsequently granted accelerated approval to the BCMA-targeting BsAb Linvoseltamab in July 2025 (37). Teclistamab, another BCMA-targeting BsAb, was the only agent with an active (not recruiting) phase III trial (38) and subsequently received FDA approval in combination with daratumumab hyaluronidase-fihj in March 2026 (39).

**Figure 2:**
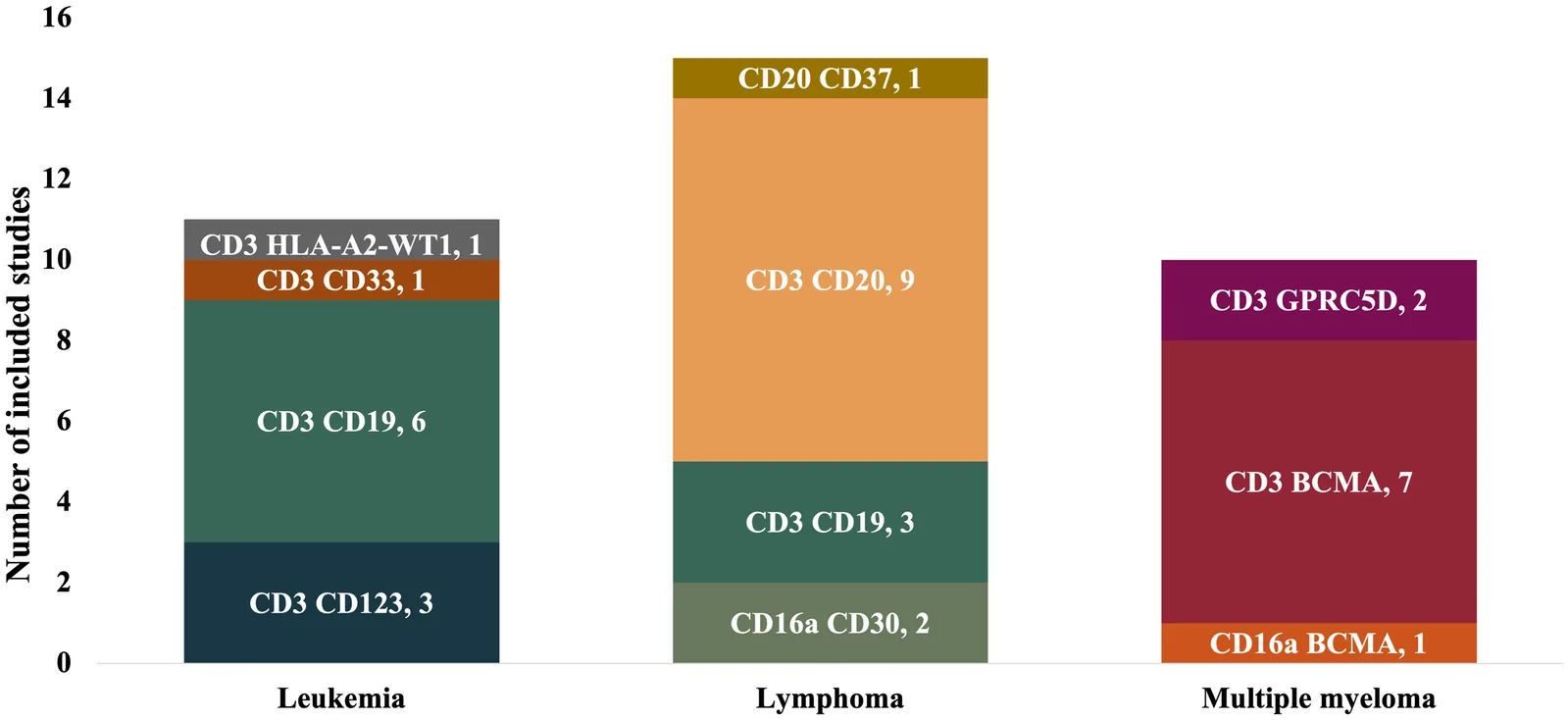
The distribution of included studies on BsAbs developed for the treatment of leukemia, lymphoma, and multiple myeloma.

**Figure 3:**
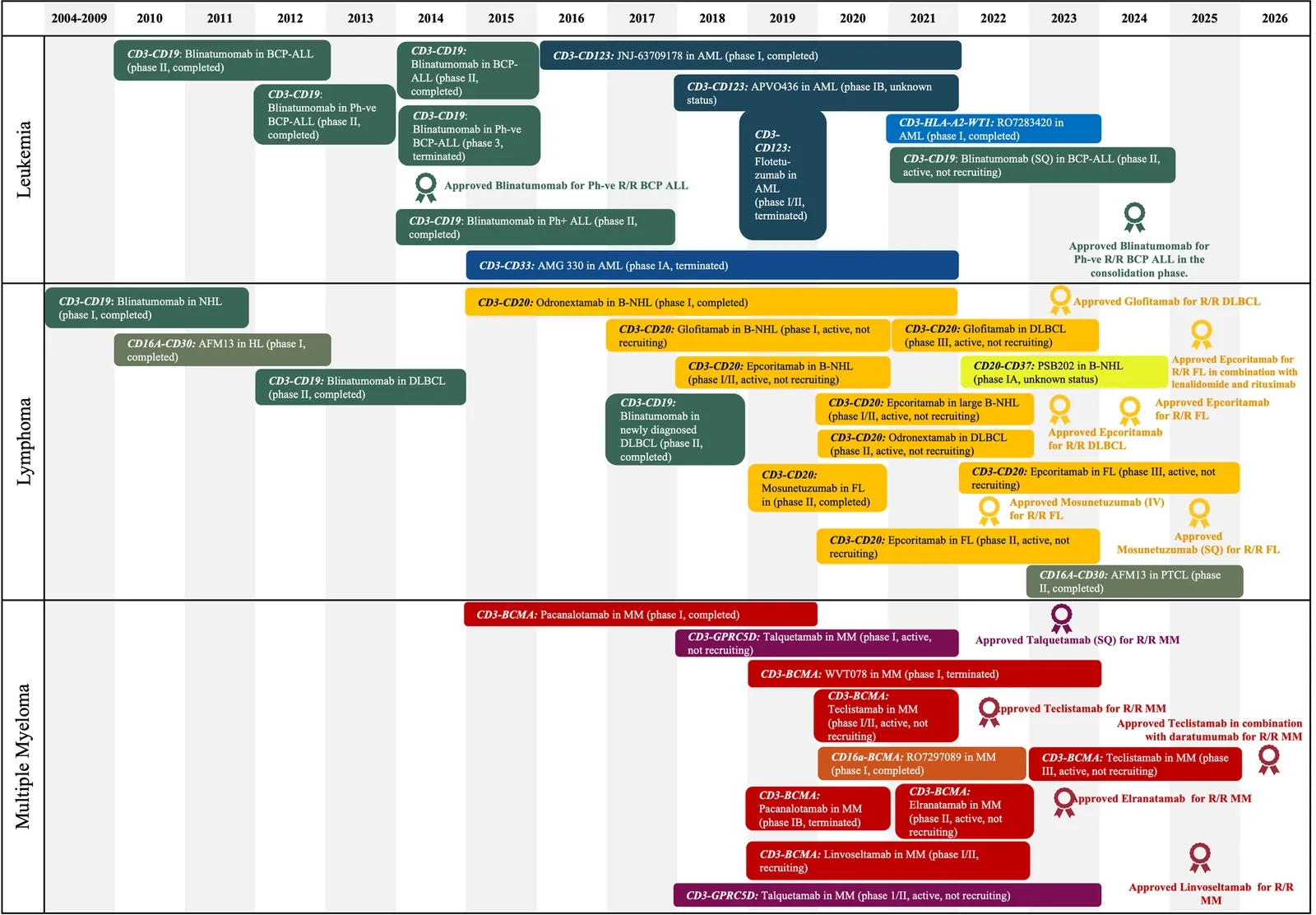
The BsAbs development timeline.

Collectively, these trends highlighted a dynamic and rapidly evolving landscape in which BsAbs have established a prominent role in ALL, B-cell lymphoma, and multiple myeloma, while clinical development in AML, HL, and PTCL remains ongoing. Details of the BsAbs distribution and their development timelines were presented in **Figures 2**, **3**.

### 3.3 Efficacy analysis

Thirty-four studies were included in the efficacy analysis, excluding those by Boyiadzis M et al. (31) and Plesner T, et al. (40) due to the absence of reported efficacy data. A comprehensive summary of the efficacy data is presented in **Table 2** for leukemia, **Table 3** for lymphoma, and **Table 4** for multiple myeloma.

**Table 2:** Efficacy data of BsAbs in leukemia.

No.	First author, year	BsAb Name	BsAb target	Sponsor	Cancer type	%MRD response	%HSCT	%ORR	%CR	Median DOR (months)	PFS (months)	Median OS (months)	Ref.
1	Topp MS., 2014	Blinatumomab	CD3-CD19	Amgen	BCP-ALL	88.00% (22/25)	52.00% (13/25)	69.44% (25/36)	41.67% (15/36)	NR	7.60	9.80	(41)
2	Topp MS., 2015	Blinatumomab	CD3-CD19	Amgen	Ph-ve BCP-ALL	82.19% (60/73)	39.50% (32/81)	NR	42.86% (81/189)	NR	5.90	6.10	(42)
3	Kantarjian H., 2017	Blinatumomab	CD3-CD19	Amgen	Ph-ve BCP-ALL	76.00% (NR/NR)	24.00% (NR/NR)	NR	33.58% (91/271)	NR	NR	7.70	(27)
4	Gökbuget N., 2018	Blinatumomab	CD3-CD19	Amgen	BCP-ALL	77.87% (88/113)	67.27% (74/110)	NR	NR	NR	18.90	36.50	(44)
5	Martinelli G., 2021	Blinatumomab	CD3-CD19	Amgen	Ph+ ALL	87.50% (14/16)	20.00% (9/45)	35.56% (16/45)	31.10% (14/45)	6.80	6.80	9.00	(43)
6	Jabbour E., 2025	Blinatumomab (SQ)	CD3-CD19	Amgen	BCP-ALL	70.45% (62/88)	32.95% (29/88)	77.27% (68/88)	64.00% (56/88)	NR	5.00	NR	(28)
7	Uckun FM., 2021	APVO436	CD3-CD123	Aptevo	AML	NR	NR	NR	NR	NR	NR	5.93	(29)
8	Uy GL., 2021	Flotetuzumab	CD3-CD123	MacroGenics	AML	NR	NR	13.64% (12/88)	11.36% (10/88)	6.90	NR	3.20	(30)
10	Ravandi F., 2024	AMG 330	CD3-CD33	Amgen	AML	NR	25.00% (1/4)	NR	5.00% (3/60)	1.28	NR	NR	(32)
11	Hutchings M., 2025	RO7283420	CD3-HLA-A2-WT1	Hoffmann-La Roche	AML	NR	2.38% (1/42)	NR	7.46% (2/42)	NR	NR	NR	(33)

BsAb, Bispecific antibody; CD, Cluster of Differentiation; HLA-A2,WT1, Human leukocyte antigen-A2–restricted Wilms tumor 1; MRD, Measurable residual disease; HSCT, Hematopoietic stem cell transplantation; ORR, Objective response rate; CR, Complete response; DOR, Duration of response; PFS, Progression-free survival; OS, Overall survival; BCP-ALL, B-cell precursor acute lymphoblastic leukemia; Ph-ve, Philadelphia-chromosome negative; Ph+, Philadelphia chromosome-positive; ALL, acute lymphoblastic leukemia; AML, Acute myeloid leukemia; SQ, Subcutaneous; NR, Not reported.

**Table 3:** Efficacy data of BsAbs in lymphoma.

No.	First author, year	BsAb Name	BsAb target	Sponsor	Cancer type	% ORR	% CR	Median DOR (months)	PFS (months)	Median OS (months)	Ref.
12	Rothe A., 2015	AFM13	CD16A-CD30	Affimed	HL	10.71% (3/28)	NR	NR	5.00	NR	(64)
13	Goebeler ME., 2016	Blinatumomab	CD3-CD19	Amgen	NHL	22.86% (8/35)	68.57% (24/35)	13.46	NR	NR	(65)
14	Hutchings M., 2021	Glofitamab	CD3-CD20	Hoffmann-La Roche	B-NHL	53.80% (92/171)	36.84% (63/171)	NR	NR	NR	(59)
15	Hutchings M., 2021	Epcoritamab	CD3-CD20	Genmab	B-NHL	22.06% (15/68)	14.71% (10/68)	NR	NR	NR	(60)
16	Bannerji R., 2022	Odronextamab	CD3-CD20	Regeneron	B-NHL	49.66% (72/145)	35.86% (52/145)	NR	NR	NR	(61)
17	Wang L., 2026	PSB202	CD20-CD37	Noncommunicable Chronic Diseases-National Science and Technology Major Project	B-NHL	30.00% (3/10)	10.00% (1/10)	NR	NR	NR	(34)
18	Thieblemont C., 2022	Epcoritamab	CD3-CD20	Genmab	Large B-NHL	63.06% (99/157)	38.85% (61/157)	12.00	4.40	NR	(45)
19	Viardot A., 2016	Blinatumomab	CD3-CD19	Amgen	DLBCL	36.00% (9/25)	16.00% (4/25)	11.60	3.70	5.00	(62)
20	Katz DA., 2022	Blinatumomab	CD3-CD19	Amgen	DLBCL*	89.30% (25/28)	85.71% (24/28)	NR	NR	NR	(26)
21	Abramson JS., 2024	Glofitamab	CD3-CD20	Hoffmann-La Roche	DLBCL	68.31% (125/183)	58.47% (107/183)	NR	13.8	25.5	(46)
22	Kim WS., 2025	Odronextamab	CD3-CD20	Regeneron	DLBCL	51.97% (66/127)	31.5% (40/127)	10.20	4.40	9.20	(47)
23	Budde LE., 2022	Mosunetuzumab	CD3-CD20	Genentech	FL	80.00% (72/90)	60.00% (54/90)	22.80	17.90	NR	(48)
24	Linton KM., 2024	Epcoritamab	CD3-CD20	Genmab	FL	82.03% (105/128)	62.50% (80/128)	18.00	NR	NR	(49)
25	Falchi L., 2026	Epcoritamab	CD3-CD20	AbbVie and Genmab.	FL	95.06% (231/243)	82.72% (201/243)	NR	NR	NR	(50)
26	Kim WS., 2025	Acimtamig	CD16A-CD30	Affimed	PTCL	32.41% (35/108)	10.19% (11/108)	2.30	3.50	13.8	(63)

*Newly diagnosed DLBCL. BsAb, Bispecific antibody; CD, Cluster of Differentiation; ORR, Objective response rate; CR, Complete response; DOR, Duration of response; PFS, Progression-free survival; OS, Overall survival; HL, Hodgkin lymphoma; NHL, Non-Hodgkin lymphoma; B-NHL, B-cell non-Hodgkin lymphoma; DLBCL, Diffuse large B-cell lymphoma; FL, Follicular lymphoma; PTCL, Peripheral T-cell lymphoma; NR, Not reported.

**Table 4:** Efficacy data of BsAbs in multiple myeloma.

No.	First author, year	BsAb Name	BsAb target	Sponsor	Cancer type	%MRD response	% ORR	% CR	% sCR	% VGPR	Median DOR (months)	PFS (months)	Median OS (months)	Ref.
27	Topp MS., 2020	Pacanalotamab	CD3-BCMA	Boehringer Ingelheim	MM	14.29% (6/42)	30.95% (13/42)	21.43% (9/42)	NR	4.76% (2/42)	8.40	NR	NR	(51)
28.1	Chari A., 2022	Talquetamab (SQ)	CD3-GPRC5D	Janssen	MM	NR	NR	4.63% (5/108)	15.74% (17/108)	32.41% (35/108)	11.90	NR	NR	(58)
28.2	Chari A., 2022	Talquetamab (IV)	CD3-GPRC5D	Janssen	MM	NR	NR	11.11% (2/18)	16.67% (3/18)	33.33% (3/18)	10.90	NR	NR	(58)
29	Moreau P., 2022	Teclistamab	CD3-BCMA	Janssen	MM	26.67% (44/165)	63.03% (104/165)	6.67% (11/165)	32.73% (54/165)	19.39% (32/165)	18.40	11.30	18.30	(52)
30	Raab MS., 2023	WVT078	CD3-BCMA	Novartis	MM	7.69% (2/26)	30.30% (10/26)	9.09% 7.69% (2/26)	3.85% (1/26)	11.54% (3/26)	7.60	NR	NR	(53)
31	Lesokhin AM., 2023	Elranatamab	CD3-BCMA	Pfizer	MM	60.47% (26/43)	60.98% (75/123)	19.51% (24/123)	15.45% (19/123)	21.14% (26/123)	NR	NR	NR	(54)
33	Bumma N., 2024	Linvoseltamab	CD3-BCMA	Regeneron	MM	92.86% (26/28)	70.94% (83/117)	5.13% (6/117)	44.44% (52/117)	13.68% (16/117)	29.40	NR	31.40	(55)
34	Rodriguez C., 2025	Pacanalotamab	CD3-BCMA	Amgen	MM	8.70% (2/23)	34.78% (8/23)	8.70% (2/23)	4.35% (1/23)	NR	5.50	2.83	16.00	(56)
35	Chari A., 2025	Talquetamab	CD3-GPRC5D	Janssen	MM	NR	69.48% (107/154)	10.39% (16/154)	29.87% (46/154)	18.83% (29/154)	16.90	11.20	NR	(57)
36	Touzeau C., 2026	Teclistamab	CD3-BCMA	Janssen	MM	NR	84.46% (250/296)	12.16% (36/296)	53.72% (159/296)	14.19% (42/296)	NR	NR	NR	(38)

BsAb, Bispecific antibody; CD, Cluster of Differentiation; BCMA, B-cell maturation antigen; GPRC5D, G protein-coupled receptor, class C group 5 member D; MRD, Measurable residual disease; ORR, Objective response rate; CR, Complete response; sCR, Stringent complete remission; VGPR, Very good partial response; DOR, Duration of response; PFS, Progression-free survival; OS, Overall survival; MM, Multiple myeloma; SQ, Subcutaneous; IV, Intravenous; NR, Not reported.

BsAbs demonstrated substantial clinical activity in ALL, B-cell lymphomas, and multiple myeloma. However, considerable heterogeneity in response rates, response depth, and durability was observed across studies. In contrast, therapeutic activity in AML, HL, and PTCL remained limited.

#### 3.3.1 Leukemia

In R/R BCP-ALL, Blinatumomab demonstrated substantial clinical activity, with CR rates ranging from 31.11% to 64.00% (27, 28, 41–43). MRD response rates were consistently high, ranging from 70.45% to 88.00% (27, 28, 41–44), including in the study evaluating the subcutaneous formulation (28). HSCT bridging rates ranged from 20.00% to 67.27% (27, 28, 41–44). Median PFS and median OS of R/R BCP-ALL patients receiving Blinatumomab ranged from 5.00 to 18.90 months (28, 41–44) and 6.10 to 36.50 months (27, 41–44) respectively. Across studies, higher HSCT bridging rates were generally reported alongside longer PFS and OS, although this observation should be interpreted cautiously given differences in study populations and designs. Data in Philadelphia chromosome-positive (Ph+) ALL remain more limited and should be interpreted in the context of distinct disease biology, prior therapy, and the increasing use of tyrosine kinase inhibitors (TKIs) in combination strategies. In the included Ph+ ALL study, Blinatumomab achieved an MRD negativity rate of 87.50%, with an HSCT bridging rate of 20.00%, median PFS of 6.8 months, and median OS of 9.0 months (43).

In AML, BsAbs targeting CD3–CD123, CD3–CD33, and CD3–HLA-A2–WT1 demonstrated preliminary antileukemic activity, with HSCT rates of 2.38% to 25.00% (32, 33), CR rates of 5.00% to 11.36% (30, 32, 33), and median OS ranging from 3.2 to 5.93 months (29, 30).

#### 3.3.2 Lymphoma

In B-NHL, CD3–CD20 BsAbs demonstrated substantial antitumor activity, although response rates varied by lymphoma subtype. In R/R DLBCL or large B-cell lymphoma, CD3–CD20 BsAbs demonstrated clinically meaningful activity, with ORRs ranging from 51.97% to 68.31% (45–47) and CR rates from 31.50% to 58.47% (45–47). Median DoR ranged from 10.20 to 12.00 months (45, 47), median PFS from 4.40 to 13.80 months (45–47), and median OS from 9.20 to 25.50 months (46, 47). In R/R FL, ORRs ranged from 80.00% to 95.06% (48–50) and CR rates from 60.00% to 82.72% (48–50). Median DoR ranged from 18.00 to 22.80 months (48, 49), while median PFS was reported as 17.90 months in one study (48).

#### 3.3.3 Multiple myeloma

In multiple myeloma, CD3–BCMA and CD3–GPRC5D BsAbs demonstrated substantial clinical activity, with ORRs ranging from 30.95% to 84.46% (38, 51–57). Deeper responses were also observed, with VGPR rates ranging from 4.76% to 32.41% (38, 51–55, 57, 58) and stringent complete remission (sCR) rates from 3.85% to 53.72% (38, 52–58). Among studies reporting MRD, MRD response rates ranged from 7.69% to 92.86% (51–56). Median DoR ranged from 5.50 to 29.40 months (51–53, 55–58), while median PFS and median OS ranged from 2.83 to 11.30 months (51, 56, 57) and 16.00 to 31.40 months (52, 55, 56), respectively.

### 3.4 Safety analysis

Safety remains a major challenge in BsAb therapy across hematologic malignancies. The toxicity profile varied according to BsAb construct, target antigen, disease type, and dosing strategy.

#### 3.4.1 Overall safety profile

Across studies, SAEs ranged from 12.17% to 91.30% (26–29, 31–34, 38, 41–43, 45–52, 56–63), while grade ≥3 adverse events occurred frequently in all hematologic malignancies, ranging from 27.42% to 95.65% (26–28, 31–34, 38, 40–50, 52–56, 58, 59, 61–64). Despite these toxicities, treatment discontinuation generally remained below 20% (26–32, 34, 38, 40–45, 47–56, 58–61, 63–65), and treatment-related mortality was uncommon. Exceptions included a phase I CD3–HLA-A2–WT1 BsAb study in AML and a phase III Glofitamab study in DLBCL, which reported treatment discontinuation rates of 34.21% (33) and 57.92% (46), respectively. Overall, severe adverse events were frequent, whereas treatment discontinuation and treatment-related mortality remained low.

#### 3.4.2 Cytokine release syndrome

CRS was the most common immune-mediated toxicity of CD3-engaging BsAbs. Across studies, CRS incidences ranged from 1.59% to 82.61% (26–33, 38, 41–61). Most CRS events were grade 1–2, developed within 1 to 2 days (28, 31–33, 45, 48–50, 52, 54, 55, 58–60) after treatment initiation, and resolved within 2 to 3 days (28, 32, 33, 38, 45, 48–50, 52, 54, 55, 58–61). Tocilizumab and corticosteroids were commonly used for CRS management, particularly in studies incorporating step-up dosing schedules.

A CRS incidence of 7.41% was reported in a phase I study of the CD16A-engaging BsAb RO7297089 (CD16A–BCMA) (40). However, this finding was derived from a single small early-phase study (n = 27), and CRS was not reported for the CD16A–CD30 BsAb AFM13 in patients with HL (64) and PTCL (63).

#### 3.4.3 Neurotoxicity

Neurologic adverse events were reported across all hematologic malignancies treated with CD3-engaging BsAbs, although their incidence varied considerably. Overall, neurologic adverse events ranged from 0.41% to 71.05% (26–29, 38, 41–48, 50, 52, 54, 56, 58–62, 65), whereas ICANS remained relatively uncommon (0.00%–21.59%) (28, 33, 45, 46, 48, 49, 51, 52, 54, 55, 57, 59, 61) and was reported more consistently in recent studies adopting standardized immune effector cell-associated neurotoxicity grading criteria.

Among leukemia studies, neurologic adverse events were reported in up to 62.22% of patients receiving intravenous Blinatumomab (27, 28, 41–44), whereas ICANS was observed only with the subcutaneous formulation (28).

In the CD16A-engaging BsAb RO7297089, no neurologic adverse events (0.00%) were reported (40). Neurologic outcomes, including ICANS, were not reported in the CD16A–CD30 AFM13 studies (63, 64).

#### 3.4.4 Hematologic toxicities

CD3-engaging BsAbs were associated with cytopenias, including grade ≥3 anemia ranging from 3.45% to 37.40% (27–30, 38, 42, 44, 45, 47–50, 52–61, 65), neutropenia ranging from 3.41% to 68.72% (26–28, 30, 38, 41, 42, 44, 45, 47–50, 52–62, 65), and thrombocytopenia ranging from 2.17% to 23.58% (27–30, 38, 41, 42, 44, 45, 47–50, 52–54, 56–62, 65). In multiple myeloma, hematologic toxicities were frequently reported, particularly among heavily pretreated patients receiving CD3-BCMA BsAbs, with grade ≥3 neutropenia (13.04% to 64.24%) (38, 52–56), anemia (17.87% to 37.40%) (38, 52–56), and thrombocytopenia (10.65% to 23.58%) (38, 52–54, 56). Non-CD3 BsAbs demonstrated variable hematologic toxicities. CD16A-engaging agents reported grade ≥3 anemia ranging from 3.70% to 29.63% (40, 63, 64), thrombocytopenia ranging from 1.85% to 22.22% (40, 63, 64), and neutropenia of 7.41% (63), while a CD20-CD37 BsAbs reported grade ≥3 neutropenia of 60.00% (34).

#### 3.4.5 Infections

Infectious complications were commonly reported with CD3-engaging BsAbs, including serious infections (1.30% to 37.10%) (26, 27, 29, 31, 38, 41, 46–49, 51, 53, 54, 56–59, 61, 62, 65), grade ≥3 infections (2.17% to 76.36%) (26, 27, 29, 38, 41, 45, 47, 48, 50–52, 54–57, 59, 61, 62, 65), opportunistic infections (0.53% to 18.11%) (26, 27, 38, 41, 42, 45, 47, 49–51, 53–55, 57–59, 61, 65), and hypogammaglobulinemia (1.82% to 69.10%) (27, 38, 41, 52, 65). In multiple myeloma, BCMA-targeting BsAbs reported frequent infection-related toxicities, including serious infections (4.35% to 33.33%) (38, 51, 53, 54, 56), grade ≥3 infections (8.70% to 76.36%) (38, 51, 52, 54–56), opportunistic infections (3.03% to 15.45%) (38, 51, 53–55), and hypogammaglobulinemia 69.10% in the phase III Teclistamab study (38). Limited data were available for non-CD3 BsAbs, with CD16A-engaging agents reporting serious infections (3.57%), grade ≥3 infections (21.43%), and opportunistic infections (3.57%) (64), with no reported hypogammaglobulinemia (40, 63, 64).

A detailed summary of the safety data is provided in **Table 5**, with additional safety data presented in **Supplementary Material 4**.

**Table 5:** Safety data of BsAbs in leukemia, lymphoma, and multiple myeloma.

No.	First author, year	BsAb Name	BsAb target	Sponsor	Cancer type	SAEs	AEs ≥ 3 AEs	CRS (all grades)	Neuro-toxic (all grades)	Anemia (≥ 3 AEs)	Neutro-penia (≥ 3 AEs)	Thrombo-cytopenia (≥ 3 AEs)	Serious infec-tions	Infection (≥ 3 AEs)	Ref.
Leukemia															
1	Topp MS., 2014	Blinatumomab	CD3-CD19	Amgen	BCP-ALL	66.67% (24/36)	65.22% (15/23)	5.56% (2/36)	16.67% (6/36)	NR	11.11% (4/36)	11.11% (4/36)	33.33% (12/36)	13.89% (5/36)	(41)
2	Topp MS., 2015	Blinatumomab	CD3-CD19	Amgen	Ph-ve BCP-ALL	12.17% (23/189)	82.01% (155/189)	1.59% (3/189)	51.85% (98/189)	14.29% (27/189)	15.87% (30/189)	8.47% (16/189)	NR	NR	(42)
3	Kantarjian H.,2017	Blinatumomab	CD3-CD19	Amgen	Ph-ve BCP-ALL	61.8% (165/267)	86.52% (231/267)	14.23% (38/267)	9.36% (25/267)	25.84% (69/267)	37.83% (101/267)	17.60% (47/267)	28.09% (75/267)	34.08% (91/267)	(27)
4	Gökbuget N., 2018	Blinatumomab	CD3-CD19	Amgen	BCP-ALL	NR	59.48% (69/116)	3.45% (4/116)	52.59% (61/116)	3.45% (4/116)	15.52% (18/116)	4.31% (5/116)	NR	NR	(44)
5	Martinelli G., 2021	Blinatumomab	CD3-CD19	Amgen	Ph+ ALL	62.22% (28/45)	84.44% (38/45)	8.89% (4/45)	62.22% (28/45)	NR	NR	NR	NR	NR	(43)
6	Jabbour E., 2025	Blinatumomab (SQ)	CD3-CD19	Amgen	BCP-ALL	79.55% (70/88)	78.41% (69/88)	72.73% (64/88)	13.64% (12/88)	13.64% (12/88)	21.59% (19/88)	12.5% (11/88)	NR	NR	(28)
7	Uckun FM., 2021	APVO436	CD3-CD123	Aptevo	AML	28.26% (13/46 )	NR	21.74% (10/46)	10.87% (5/46)	4.35% (2/46)	NR	2.17% (1/46)	2.17% (1/46)	2.17% (1/46)	(29)
8	Uy GL., 2021	Flotetuzumab	CD3-CD123	Macro-Genics	AML	NR	NR	80.68% (71/88)	NR	28.41% (25/88)	3.41% (3/88)	20.45% (18/88)	NR	NR	(30)
9	Boyiadzis M., 2023	JNJ-63709178	CD3-CD123	Janssen	AML	64.52% (40/62)	70.97% (44/62)	43.55% (27/62)	NR	NR	NR	NR	37.1% (23/62)	NR	(31)
10	Ravandi F., 2024	AMG 330	CD3-CD33	Amgen	AML	38.96% (30/77)	93.51% (72/77)	77.92% (60/77)	NR	NR	NR	NR	NR	NR	(32)
11	Hutchings M., 2025	RO7283420	CD3-HLA-A2-WT1	Hoffmann-La Roche	AML	77.42% (48/62)	27.42% (17/62)	61.29% (38/62)	NR	NR	NR	NR	NR	NR	(33)
Lymphoma															
12	Rothe A., 2015	AFM13	CD16A-CD30	Affimed	HL	NR	28.57% (8/28)	NR	NR	7.14% (2/28)	NR	3.57% (1/28)	3.57% (1/28)	21.43% (6/28)	(64)
13	Goebeler ME., 2016	Blinatumomab	CD3-CD19	Amgen	NHL	NR	NR	NR	71.05% (54/76)	6.58% (5/76)	17.11% (13/76)	11.84% (9/76)	10.53% (8/76)	50.00% (38/76)	(65)
14	Hutchings M, 2021	Glofitamab	CD3-CD20	Hoffmann-La Roche	B-NHL	58.48% (100/171)	56.73% (97/171)	50.29% (86/171)	43.27% (74/171)	7.60% (13/171)	25.15% (43/171)	8.19% (14/171)	17.54% (30/171)	51.46% (88/171)	(59)
15	Hutchings M, 2021	Epcoritamab	CD3-CD20	Genmab	B-NHL	67.65% (46/68)	NR	58.82% (40/68)	5.88% (4/68)	13.24% (9/68)	14.71% (10/68)	5.88% (4/68)	NR	NR	(60)
16	Bannerji R., 2022	Odronextamab	CD3-CD20	Regene-ron	B-NHL	61.38% (89/145)	82.07% (119/145)	61.38% (90/145)	12.41% (18/145)	24.83% (36/145)	18.62% (27/145)	13.79% (20/145)	6.21% (9/145)	22.76% (33/145)	(61)
17	Wang L., 2026	PSB202	CD20-CD37	Noncommunicable Chronic Diseases-National Science and Technology Major Project	B-NHL	26.67% (4/15)	60.00% (9/15)	0.00% (0/15)	0.00% (0/15)	6.67% (1/15)	60.00% (9/15)	33.33% (5/15)	NR	40.00% (6/15)	(34)
18	Thieblemont C., 2022	Epcoritamab	CD3-CD20	Genmab	Large B- NHL	56.69% (89/157)	61.15% (96/157)	49.68% (78/157)	0.64% (1/157)	10.19% (16/157)	14.65% (23/157)	5.73% (9/157)	NR	14.65% (23/157)	(45)
19	Viardot A., 2016	Blinatumomab	CD3-CD19	Amgen	DLBCL	91.30% (21/23)	95.65% (22/23)	NR	69.57% (16/23)	NR	17.39% (4/23)	17.39% (4/23)	21.74% (5/23)	13.04% (3/23)	(62)
20	Katz DA., 2022	Blinatumomab	CD3-CD19	Amgen	DLBCL*	25.00% (7/28)	32.14% (9/28)	17.86% (5/28)	60.71% (17/28)	NR	14.29% (4/28)	NR	3.57% (1/28)	10.71% (3/28)	(26)
21	Abramson JS., 2024	Glofitamab	CD3-CD20	Hoffmann-La Roche	DLBCL	54.44% (98/180)	77.78% (140/180)	44.00% (76/172)	30.56% (55/180)	NR	NR	NR	25.56% (46/180)	NR	(46)
22	Kim WS., 2025	Odronextamab	CD3-CD20	Regeneron	DLBCL	64.57% (82/127)	84.25% (107/127)	55.00% (70/127)	42.52% (54/127)	22.83% (29/127)	25.98% (33/127)	14.96% (19/127)	11.81% (15/127)	38.58% (49/127)	(47)
23	Budde LE., 2022	Mosunetuzu-mab	CD3-CD20	Genentech	FL	46.67% (42/90)	51.11% (46/90)	44.44% (40/90)	5.56% (5/90)	7.78% (7/90)	26.67% (24/90)	4.44% (4/90)	20.00% (18/90)	14.44% (13/90)	(48)
24	Linton KM., 2024	Epcoritamab	CD3-CD20	Genmab	FL	46.88% (60/128)	37.50% (48/128)	66.41% (85/128)	NR	6.25% (8/128)	24.22% (31/128)	5.47% (7/128)	7.81% (10/128)		(49)
25	Falchi L., 2026	Epcoritamab	CD3-CD20	AbbVie and Genmab.	FL	55.56% (135/243)	90.12% (219/243)	35.00% (85/243)	0.41% (1/243)	7.82% (19/243)	68.72% (167/243)	9.47% (23/243)	NR	33.33% (81/243)	(50)
26	Kim WS., 2025	Acimtamig	CD16A-CD30	Affimed	PTCL	39.81% (43/108)	53.70% (58/108)	NR	NR	3.7% (4/108)	7.41% (8/108)	1.85% (2/108)	NR	NR	(63)
Multiple myeloma															
27	Topp MS., 2020	Pacanalotamab	CD3-BCMA	Boehringer Ingelheim	MM	47.62% (20/42)	NR	38.09% (16/42)	NR	NR	NR	NR	33.33% (14/42)	19.05% (8/42)	(51)
28.1	Chari A., 2022	Talquetamab (SQ)	CD3-GPRC5D	Janssen	MM	40.76% (58/130)	86.15% (112/130)	72.31% (94/130)	7.69% (10/130)	27.69% (36/130)	45.38% (59/130)	20.00% (26/130)	1.54% (2/130)	NR	(58)
28.2	Chari A., 2022	Talquetamab (IV)	CD3-GPRC5D	Janssen	MM	34.31% (35/102)	90.19% (92/102)	49.02% (50/102)	5.88% (6/102)	33.33% (34/102)	26.47% (27/102)	12.75% (13/102)	4.90% (5/102)	NR	(58)
29	Moreau P., 2022	Teclistamab	CD3-BCMA	Janssen	MM	64.85% (107/165)	94.55% (156/165)	72.12% (119/165)	14.55% (24/165)	36.97% (61/165)	64.24% (106/165)	21.21% (35/165)	NR	76.36% (126/165)	(52)
30	Raab MS., 2023	WVT078	CD3-BCMA	Novartis	MM	NR	78.78% (26/33)	60.61% (20/33)	NR	30.30% (10/33)	18.18% (6/33)	15.15% (5/33)	12.12% (4/33)	NR	(53)
22	Plesner T., 2023	RO7297089	CD16a-BCMA	Genen-tech	MM	NR	62.96% (17/27)	7.41% (2/27)	0.00% (0/27)	29.63% (8/27)	NR	22.22% (6/27)	NR	NR	(40)
31	Lesokhin AM., 2023	Elranatamab	CD3-BCMA	Pfizer	MM	NR	70.73% (87/123)	57.72% (71/123)	30.89% (38/123)	37.40% (46/123)	48.78% (60/123)	23.58% (29/123)	6.50% (8/123)	39.84% (49/123)	(54)
33	Bumma N., 2024	Linvoseltamab	CD3-BCMA	Regeneron	MM	NR	73.5% (86/117)	46.15% (54/117)	NR	30.77% (36/117)	41.88% (49/117)	NR	NR	35.9% (42/117)	(55)
34	Rodriguez C., 2025	Pacanalotamab	CD3-BCMA	Amgen	MM	82.61% (19/23)	65.22% (15/23)	82.61% (19/23)	52.17% (12/23)	21.74% (5/23)	13.04% (3/23)	13.04% (3/23)	4.35% (1/23)	8.70% (2/23)	(56)
35	Chari A., 2025	Talquetamab	CD3-GPRC5D	Janssen	MM	52.60% (81/154)	NR	74.68% (115/154)	NR	25.97% (40/154)	21.43% (33/154)	18.18% (28/154)	1.3% (2/154)	18.18% (28/154)	(57)
36	Touzeau C., 2026	Teclistamab	CD3-BCMA	Janssen	MM	56.70% (NR/NR)	84.88% (247/291)	65.98% (192/291)	4.12% (12/291)	17.87% (52/291)	54.3% (158/291)	10.65% (31/291)	5.5% (16/291)	41.58% (121/291)	(38)

*Newly diagnosed DLBCL. BsAb, Bispecific antibody; CD, Cluster of Differentiation; BCMA, B-cell maturation antigen; GPRC5D, G protein-coupled receptor, class C group 5 member D; HLA-A2,WT1, Human leukocyte antigen-A2–restricted Wilms tumor 1; SAEs, Serious Adverse Events; ≥ 3 AEs, Adverse Events ≥ grade 3 AEs; CRS, Cytokine Release Syndrome; BCP-ALL, B-cell precursor acute lymphoblastic leukemia; Ph-ve, Philadelphia-chromosome negative; Ph+ ALL, Philadelphia chromosome-positive; ALL, Acute lymphoblastic leukemia; AML, Acute myeloid leukemia; NHL, Non-Hodgkin lymphoma; B-NHL, B-cell non-Hodgkin lymphoma; HL, Hodgkin lymphoma; DLBCL, Diffuse large B-cell lymphoma; FL, Follicular lymphoma; PTCL, Peripheral T-cell lymphoma; MM, Multiple myeloma; SQ, Subcutaneous; IV, Intravenous; NR, Not reported.

### 3.5 Quality assessment

**Figure 4:**
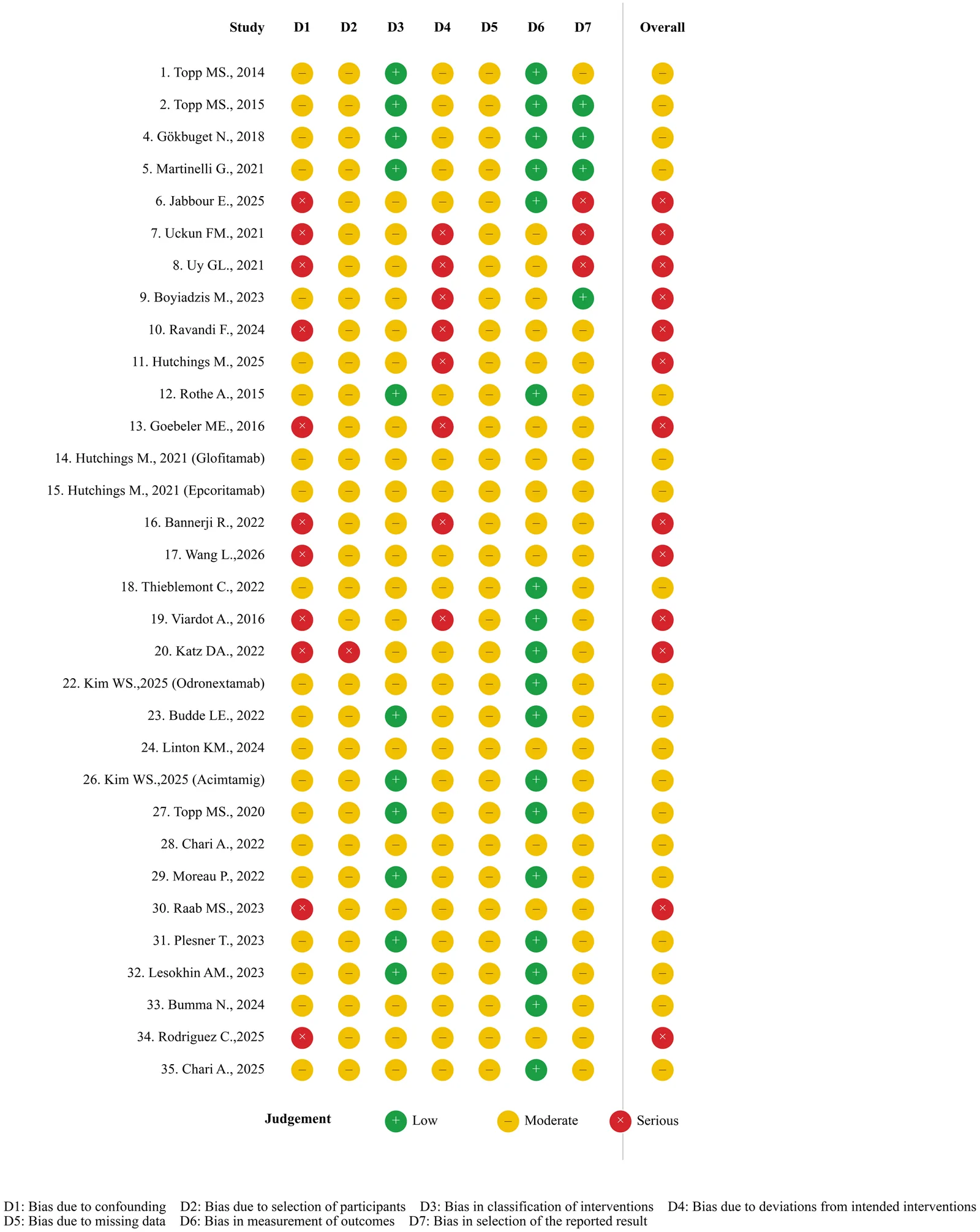
The quality assessment of phase I-II non-randomized trials by using ROBINS-I V2.

**Figure 5:**
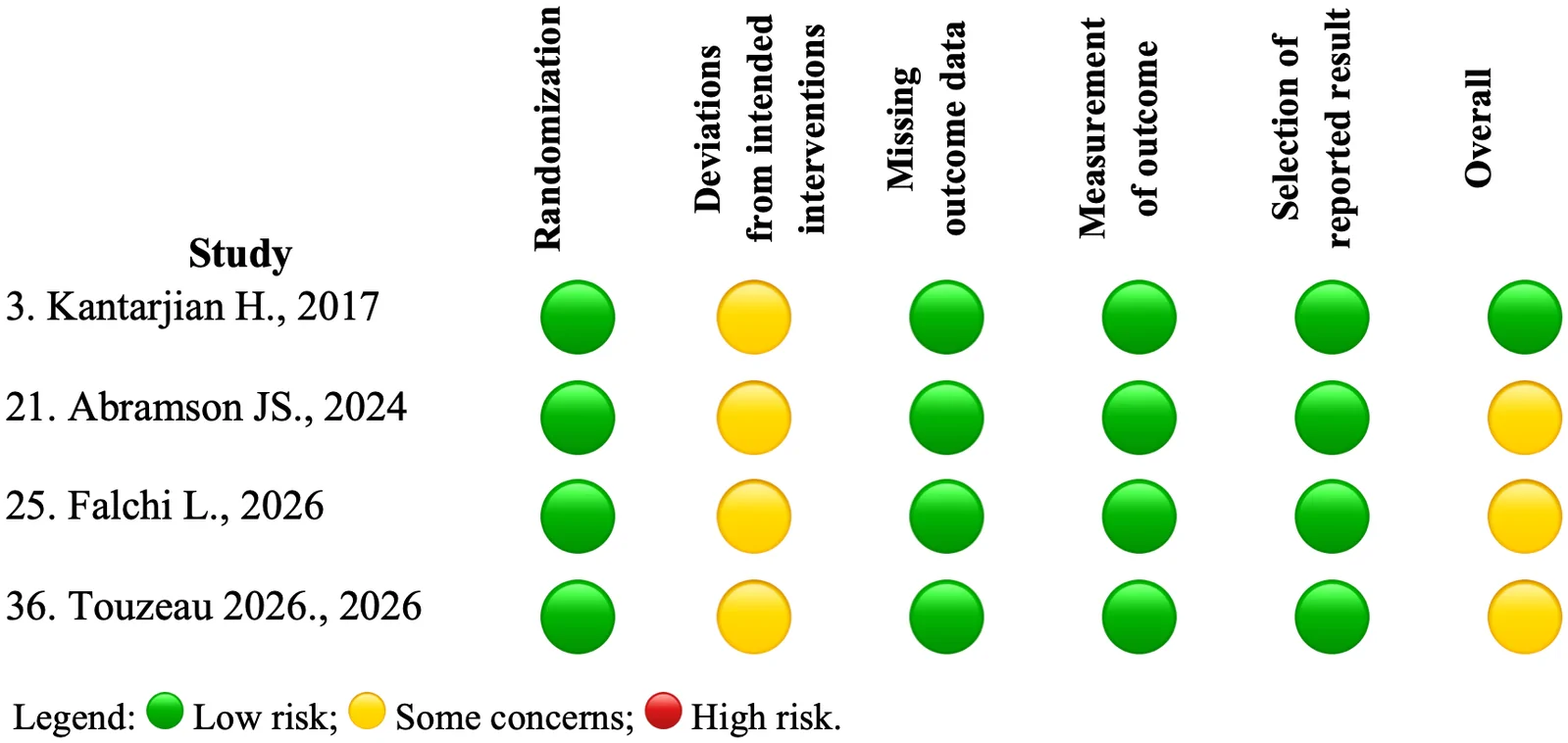
The quality assessment of phase III randomized trials by using RoB 2.

The quality assessment of the 36 included studies, comprising 32 phase I-II studies and 4 phase III randomized controlled trials, is summarized in **Figures 4**, **5**.

Briefly, all non-randomized studies (n = 32) were judged as moderate in 19 studies and serious in 13. Risk of bias due to confounding, primarily related to the predominantly single-arm, non-randomized study designs. Bias arising from participant selection was consistently rated as moderate in 31 studies, while one study was judged at serious risk. Bias due to missing data was moderate, primarily because of immature follow-up and the exclusion of non-response patients. Bias in the selection of the reported result was low in 4 studies, moderate in 25, and serious in 3 owing to selective reporting of responder analyses. Outcome measurement was judged to be at low risk in 18 studies using centrally adjudicated objective criteria but moderate in 14 owing to unblinded, investigator-assessed outcomes or variability in adverse event and cytokine release syndrome grading.

The risk of bias assessment of the 4 included phase III randomized controlled trials. One study was judged as low risk of bias, whereas the remaining three were rated as having some concerns. The primary source of concern was deviations from intended interventions due to the open-label study design. No study was judged to be at high risk of bias.

## 4 Discussion

This systematic review synthesized the current evidence on the clinical development, efficacy, safety, and regulatory landscape of bispecific antibodies across hematologic malignancies. Overall, the evidence demonstrated substantial clinical progress, particularly in B-cell malignancies where multiple agents have achieved regulatory approval. In contrast, BsAbs targeting AML, HL, and T-cell lymphoma remain in early stages of clinical development. The findings also highlight the continued expansion of therapeutic targets, together with improvements in dosing strategies and toxicity management, reflecting the rapid evolution of the BsAb field over the past decade.

Over the years, BsAb development has expanded progressively from CD19-directed therapy in BCP-ALL to multiple disease-specific targets across hematologic malignancies. Clinical development subsequently expanded to CD20-targeting BsAbs for B-cell lymphomas and BCMA- and GPRC5D-targeting BsAbs for multiple myeloma. More recently, additional targets, including CD33, HLA-A2-WT1, and CD20-CD37, have entered early clinical development (32, 33). This development reflected the increasing diversification of therapeutic targets across hematologic malignancies.

Clinical efficacy has become well established for BsAbs in several hematologic malignancies, particularly BCP-ALL, B-cell lymphomas, and multiple myeloma. In BCP-ALL, the consistently high MRD response rates achieved with Blinatumomab support its established role in the management of the relapsed/refractory disease (27, 41, 44). In B-cell lymphomas, the availability of multiple CD20-targeting BsAbs has expanded treatment options for both DLBCL and FL (45, 48, 59), while in multiple myeloma, the rapid clinical development and regulatory approval of BCMA- and GPRC5D-targeting BsAbs have further broadened therapeutic options for heavily pretreated patients (52, 55, 58). In contrast, the clinical development of BsAbs for AML remains less mature, with most candidates still in early-phase clinical evaluation (29, 32, 33).

At the same time, clinical development is increasingly focused on evaluating BsAbs in combination regimens and earlier treatment settings, aiming to improve the depth and durability of response while overcoming resistance associated with heavily pretreated disease (38, 46, 50). BsAbs have been integrated into the treatment landscape of hematologic malignancies. Current guidelines recommend Blinatumomab for MRD-positive and relapsed B-cell ALL and, in Philadelphia chromosome-positive ALL, in combination with tyrosine kinase inhibitors (TKIs) (66). CD3-CD20 and BCMA- or GPRC5D-targeting BsAbs are also recommended for relapsed/refractory lymphoma and multiple myeloma after prior therapies, alongside CAR-T therapy in appropriate patients (67, 68). Compared with CAR-T therapy, BsAbs offer off-the-shelf availability, rapid treatment initiation, and outpatient feasibility, whereas CAR-T therapy may provide more durable responses in selected patients (69, 70). Optimal sequencing should consider prior antigen-directed therapies, including CAR-T therapy and antibody-drug conjugates, T-cell fitness, infection risk, durability of response, transplant eligibility, and patient preferences, and remains an area of ongoing investigation (71, 72).

An important observation from this review is that BsAbs targeting the same antigen may demonstrate different clinical outcomes. For example, variability in efficacy was observed among CD20-targeting BsAbs in B-cell lymphomas and among BCMA-targeting BsAbs in multiple myeloma, despite sharing the same therapeutic target. These differences are unlikely to be explained by target selection alone and may instead reflect differences in molecular architecture, epitope recognition, binding valency, route of administration, dosing strategies, and study populations (73–75). In addition, differences in disease characteristics, prior treatment exposure, and trial design further limit direct comparisons across studies. Consequently, efficacy should be interpreted within the context of individual clinical trials rather than through indirect cross-trial comparisons.

CD16A-engaging BsAbs act through natural killer cell–mediated cytotoxicity (76). The evidence for this class was derived from small, single-arm, early-phase studies with short follow-up and incomplete efficacy and safety reporting (40, 63). Therefore, the findings of the present review should be interpreted cautiously, as differences in disease setting, patient population, dosing strategy, and CRS grading criteria preclude direct comparisons with CD3-engaging BsAbs. Larger prospective studies with longer follow-up are needed to better define their efficacy, durability, and safety.

In parallel with the progressive expansion of therapeutic targets, the clinical implementation of BsAbs has also evolved substantially. Early BsAb therapy was limited by the need for continuous intravenous infusion and concerns regarding immune-mediated toxicities, particularly cytokine release syndrome (CRS) and neurologic adverse events. More recently, step-up dosing schedules, subcutaneous formulations, and fixed-duration treatment strategies have been incorporated into clinical development to improve treatment administration and safety management (75, 77, 78). In the present review, CRS was predominantly grade 1–2, generally occurred within 1 to 2 days after treatment initiation, resolved within 2 to 3 days, and was commonly managed with tocilizumab and corticosteroids (28, 48, 49, 52, 54, 55, 58, 59). Although grade ≥3 adverse events were frequently reported, treatment discontinuation and treatment-related mortality remained generally low across studies, suggesting that these toxicities can be effectively managed with current treatment strategies (48, 52, 58–60). Nevertheless, hematologic toxicities, infections, and hypogammaglobulinemia remain important clinical concerns, particularly with BCMA-targeting BsAbs (38), highlighting the need for continued optimization of supportive care and long-term safety monitoring.

Future studies should also continue to optimize dosing strategies, identify predictive biomarkers of response and toxicity, and evaluate long-term safety to further refine the clinical use of BsAbs.

This review has several strengths. To our knowledge, it provides one of the most comprehensive syntheses of the current BsAb landscape across hematologic malignancies by integrating evidence on clinical development, efficacy, safety, and regulatory progress. The inclusion of both approved and investigational BsAbs enabled a broad overview of the evolving therapeutic landscape and emerging targets. However, several limitations should be acknowledged. The included studies were heterogeneous with respect to study design, patient populations, prior lines of therapy, treatment regimens, and outcome reporting, precluding direct comparisons across studies and limiting quantitative synthesis. In addition, most evidence for emerging BsAbs was derived from early-phase, single-arm clinical trials with relatively short follow-up, and long-term efficacy and safety data remain limited. Future updates will be important as additional phase III studies and longer-term follow-up become available.

## 5 Conclusion

Bispecific antibodies have established clinical activity in B-cell precursor acute lymphoblastic leukemia, B-cell non-Hodgkin lymphoma, and multiple myeloma, while clinical development in acute myeloid leukemia, Hodgkin lymphoma, and T-cell lymphoma remains less mature. Across platforms, cytokine release syndrome, neurotoxicity, cytopenias, infections, and hypogammaglobulinemia remain important safety considerations. Future studies should focus on defining optimal treatment sequencing, improving the durability of response, identifying predictive biomarkers, optimizing infection prevention and toxicity management, and clarifying the role of emerging targets and non-CD3-engaging bispecific antibodies.

## Data Availability

The original contributions presented in the study are included in the article/**Supplementary Material**. Further inquiries can be directed to the corresponding author.

## Glossary

ADC: antibody-drug conjugate
MM: multiple myeloma
AE: adverse event
MRD: measurable (minimal) residual disease
ALL: acute lymphoblastic leukemia
NCCN: National Comprehensive Cancer Network
AML: acute myeloid leukemia, NHL, non-Hodgkin lymphoma
BBB: blood-brain barrier
NK: natural killer
BCMA: B-cell maturation antigen
NR: not reported
BCP-ALL: B-cell precursor acute lymphoblastic leukemia
ORR: overall (objective) response rate
BCR: B-cell receptor, OS
BiTE: bispecific T-cell engager
PFS: progression-free survival
BsAb: bispecific antibody, Ph+
CAR-T: chimeric antigen receptor T cell
Ph−: Philadelphia chromosome-negative
CD: cluster of differentiation, PICO, population-intervention-comparator-outcome
CI: confidence interval, PRISMA
CR: complete response
PROSPERO: International Prospective Register of Systematic Reviews
CRS: cytokine release syndrome
RCT: randomized controlled trial
DLBCL: diffuse large B-cell lymphoma
RFS: relapse-free survival
DoR/DOR: duration of response
RoB: risk of bias
FDA: US Food and Drug Administration
ROBINS-I: Risk Of Bias In Non-randomized Studies of Interventions
FL: follicular lymphoma
R/R: relapsed/refractory
GPRC5D: G protein-coupled receptor class C group 5 member D
SAE: serious adverse event
HL: Hodgkin lymphoma
sCR: stringent complete response
ICANS: immune effector cell-associated neurotoxicity syndrome
SQ: subcutaneous
IV: intravenous
TKI: tyrosine kinase inhibitor
mAb: monoclonal antibody
VGPR: very good partial response
MM: multiple myeloma
HSCT: hematopoietic stem cell transplantation
B-NHL: B-cell non-Hodgkin lymphoma
PTCL: Peripheral T-cell lymphoma
HLA-A2:WT1: Human leukocyte antigen-A2–restricted Wilms tumor 1.
