# Supplementary material for: Safety and efficacy of bispecific antibodies in hematologic neoplasms: a systematic review
Source: Front Immunol. 2026 Aug 11;17:1815519. doi: 10.3389/fimmu.2026.1815519 (PMC13503316; doi:10.3389/fimmu.2026.1815519)
Supplement: Supplementary file 2 [file Table2.docx]

**Supplementary material 2 Table Search terms and results.**

| **Database** | **Search Details** | **Results** |
| --- | --- | --- |
| Pubmed | (“bispecific”[All Fields] OR “bispecificity”[All Fields] OR “bispecifics”[All Fields] OR “Bifunctional”[All Fields] OR ((“bispecific”[All Fields] OR “bispecificity”[All Fields] OR “bispecifics”[All Fields]) AND (“monoclonal”[All Fields] OR “monoclonality”[All Fields] OR “monoclonally”[All Fields] OR “monoclonals”[All Fields] OR “monoclone”[All Fields] OR “monoclones”[All Fields])) OR (“antibodies, bispecific”[MeSH Terms] OR (“antibodies”[All Fields] AND “bispecific”[All Fields]) OR “bispecific antibodies”[All Fields] OR (“monoclonal”[All Fields] AND “antibodies”[All Fields] AND “bispecific”[All Fields]) OR “monoclonal antibodies bispecific”[All Fields]) OR “bi specific*”[All Fields] OR “2specific*”[All Fields] OR “2-specific”[All Fields] OR “two specific*”[All Fields] OR “double specific*”[All Fields] OR “dualspecific*”[All Fields] OR “dual specific*”[All Fields] OR “despecific*”[All Fields] OR “de specific*”[All Fields] OR “polyspecific*”[All Fields] OR “poly specific*”[All Fields] OR ((“t lymphocytes”[MeSH Terms] OR “t lymphocytes”[All Fields] OR “t cell”[All Fields]) AND “engager*”[All Fields])) AND (“antibodie”[All Fields] OR “antibodies”[MeSH Terms] OR “antibodies”[All Fields] OR “antibody s”[All Fields] OR “antibodys”[All Fields] OR “immunoglobulins”[MeSH Terms] OR “immunoglobulins”[All Fields] OR “antibody”[All Fields] OR “immunoglobulin*”[All Fields] OR (“immunoglobulins”[MeSH Terms] OR “immunoglobulins”[All Fields] OR (“globulins”[All Fields] AND “immune”[All Fields]) OR “globulins immune”[All Fields]) OR (“immunoglobulins”[MeSH Terms] OR “immunoglobulins”[All Fields] OR (“immune”[All Fields] AND “globulin”[All Fields]) OR “immune globulin”[All Fields]) OR (“immunoglobulins”[MeSH Terms] OR “immunoglobulins”[All Fields] OR (“globulin”[All Fields] AND “immune”[All Fields]) OR “globulin immune”[All Fields]) OR (“immunoglobulins”[MeSH Terms] OR “immunoglobulins”[All Fields] OR (“immune”[All Fields] AND “globulins”[All Fields]) OR “immune globulins”[All Fields])) AND (“Hematologic Neoplasms”[MeSH Terms] OR “Leukemia”[MeSH Terms] OR “Lymphoma”[MeSH Terms] OR “Multiple Myeloma”[MeSH Terms] OR “Myelodysplastic Syndromes”[MeSH Terms] OR “hematologic cancer*”[All Fields] OR “neoplasms, plasma cell”[MeSH Terms]) | 2,733 |
| Embase | (antibodies OR immunoglobulin* OR globulins, immune OR immune globulin* OR globulin, immune) AND (bispecific* OR bifunctional OR bispecific monoclona OR monoclonal antibodies, bispecific OR bi-specific* OR 2specific* OR 2-specific OR two-specific* OR double-specific* OR dualspecific* OR dual-specific* OR despecific* OR de-specific* OR polyspecific* OR poly-specific* OR t-cell engager*) AND (hematologic neoplasm* OR leukemia OR lymphoma OR multiple myeloma* OR myelodysplastic syndrome* OR hematologic cancer* OR plasma cell neoplasm*)  AND [embase]/lim NOT ([embase]/lim AND [medline]/lim) | 1,888 |
| Scopus | ( TITLE-ABS-KEY ( "Hematologic Neoplasm*" OR "Leukemia" OR "Lymphoma" OR "Multiple Myeloma*" OR "Myelodysplastic Syndrome*" OR "Hematologic Cancer*" OR "Plasma Cell Neoplasm" ) AND TITLE-ABS-KEY ( "bispecific*" OR "bifunctional" OR "bispecific monoclona" OR "monoclonal antibodies, bispecific" OR "bi-specific*'" OR "2specific*" OR "2-specific" OR "two-specific*" OR "double-specific*" OR "dualspecific*" OR "dual-specific*" OR "despecific*" OR "de-specific*" OR "polyspecific*" OR "poly-specific*" OR "t-cell engager*" ) AND TITLE-ABS-KEY ( "antibodies" OR "immunoglobulin*" OR "globulins, immune" OR "immune globulin*" OR "globulin, immune" ) ) AND ( LIMIT-TO ( DOCTYPE , "ar" ) ) | 2,365 |
| Web of Science | TS=("antibodies" OR "immunoglobulin*" OR "globulins, immune" OR "immune globulin*" OR "globulin, immune") AND TS=("bispecific*" OR "bifunctional" OR "bispecific monoclona" OR "monoclonal antibodies, bispecific" OR "bi-specific*'" OR "2specific*" OR "2-specific" OR "two-specific*" OR "double-specific*" OR "dualspecific*" OR "dual-specific*" OR "despecific*" OR "de-specific*" OR "polyspecific*" OR "poly-specific*" OR "t-cell engager*") AND TS=("bispecific*" OR "bifunctional" OR "bispecific monoclona" OR "monoclonal antibodies, bispecific" OR "bi-specific*'" OR "2specific*" OR "2-specific" OR "two-specific*" OR "double-specific*" OR "dualspecific*" OR "dual-specific*" OR "despecific*" OR "de-specific*" OR "polyspecific*" OR "poly-specific*" OR "t-cell engager*") AND TS=("Hematologic Neoplasm*" OR "Leukemia" OR "Lymphoma" OR "Multiple Myeloma*" OR "Myelodysplastic Syndrome*" OR "Hematologic Cancer*" OR "Plasma Cell Neoplasm*") | 3,861 |
