# Supplementary material for: Safety and efficacy of bispecific antibodies in hematologic neoplasms: a systematic review
Source: Front Immunol. 2026 Aug 11;17:1815519. doi: 10.3389/fimmu.2026.1815519 (PMC13503316; doi:10.3389/fimmu.2026.1815519)
Supplement: Supplementary file 3 [file Table3.docx]

**Supplementary material 3 Table Eligibility criteria.**

| **PICO** | **Inclusion criteria** | **Exclusion criteria** |
| --- | --- | --- |
| **Population (P)** | - Male and female patients aged ≥18 years with hematologic neoplasms (leukemia, lymphoma, or multiple myeloma). - Eligible irrespective of disease stage or number of previous lines of therapy. - Patients with other comorbidities or metastatic cancers were also included. | - Adult patients with solid tumors or non-hematologic malignancies. |
| **Intervention (I)** | - Bispecific antibodies (BsAbs), with clinical trials required to have at least one treatment arm receiving a BsAb. | - Bispecific antibody in CAR-T cell therapy. |
| **Comparator (C)** | - Any comparator or no comparator. |  |
| **Outcomes (O)** | - Efficacy outcomes, such as objective response rate (ORR), complete response (CR), overall survival (OS), and progression-free survival (PFS). - Safety outcomes, such as cytokine release syndrome (CRS), hematologic toxicity, neurotoxicity, and serious adverse events. - At least one efficacy and/or safety outcome reported. |  |
| **Study design** | - Clinical studies (phase I-III clinical trials) evaluating bispecific antibodies. - Full-text articles available. | • Pre-clinical studies, case reports, pre-print articles, reviews, editorials, systematic reviews, meta-analyses, commentary letters, conference proceedings, abstracts, seminar posters, trial protocols, re-analyses of previously published clinical trials, observational studies, retrospective studies, personal opinions, and book chapters.  • Studies written in languages other than English. |
