# Supplementary material for: Safety and efficacy of bispecific antibodies in hematologic neoplasms: a systematic review
Source: Front Immunol. 2026 Aug 11;17:1815519. doi: 10.3389/fimmu.2026.1815519 (PMC13503316; doi:10.3389/fimmu.2026.1815519)
Supplement: Supplementary file 4 [file Table4.docx]

**Supplementary material 4 Table Additional safety data of BsAbs in leukemia, lymphoma, and multiple myeloma.**

| **No.** | **First author** | **BsAb Name** | **BsAb target** | **Spon-sor** | **Cancer type** | **Treat-ment discon-tinuation** | **Treat-ment-related mortality** | **Tocilizu-mab/**  **steroid use** | **ICANS (all grades)** | **Oppor-tunistic infections** | **Hypo-gamma-globuli-nemia (≥ 3 AEs)** | **Step-up dosing** |
| --- | --- | --- | --- | --- | --- | --- | --- | --- | --- | --- | --- | --- |
| **Leukemia** | | | | | | | | | | | | |
| 1 | Topp MS., 2014 | Blinatumomab | CD3-CD19 | Amgen | BCP-ALL | 19.44% (7/36) | 2.78% (1/36) | NR | NR | 8.33% (3/36) | 8.33% (3/36) | Optimal schedule: 5 µg/m²/day week 1 → 15 µg/m²/day thereafter (cohort 2a) |
| 2 | Topp MS., 2015 | Blinatumomab | CD3-CD19 | Amgen | Ph-ve  BCP-ALL | 17.99% (34/189) | 1.59% (3/189) | 85.71% (162/189) | NR | 0.53% (1/189) | NR | Cycle 1: 9 µg/day (days 1–7) → 28 µg/day (days 8–28) |
| 3 | Kantarjian H.,2017 | Blinatumomab | CD3-CD19 | Amgen | Ph-ve  BCP-ALL | 12.36% (33/267) | 19.1% (51/267) | NR | NR | 2.62% (7/267) | 2.62% (7/267) | 9 µg/day (week 1 of cycle 1), then 28 µg/day thereafter |
| 4 | Gökbuget N., 2018 | Blinatumomab | CD3-CD19 | Amgen | BCP-ALL | 17.24% (20/116) | 0.86% (1/116) | 0.86% (1/116) | NR | NR | NR | Fixed dose 15 µg/m²/day × 28 days |
| 5 | Martinelli G., 2021 | Blinatumomab | CD3-CD19 | Amgen | Ph+ ALL | 6.67% (3/45) | 2.22% (1/45) | NR | NR | NR | NR | Cycle 1: 9 µg/day (week 1) → 28 µg/day (weeks 2–4); subsequent cycles: 28 µg/day throughout |
| 6 | Jabbour E., 2025 | Blinatumomab (SQ) | CD3-CD19 | Amgen | BCP-ALL | 12.5% (11/88) | NR | NR | 21.59% (19/88) | NR | NR | 250 µg QD (wk 1) → 500 µg TIW thereafter and 500 µg QD (wk 1) → 1000 µg TIW thereafter |
| 7 | Uckun FM., 2021 | APVO436 | CD3-CD123 | Aptevo | AML | 4.35% (2/46) | 2.17% (1/46) | NR | NR | NR | NR | RP2D cycle 1 step-up: 6 mcg → 12 mcg → 18 mcg → 18 mcg (weekly); subsequent cycles 18 mcg flat weekly. |
| 8 | Uy GL., 2021 | Flotetuzumab | CD3-CD123 | Macro-Genics | AML | 10.23% (9/88) | NR | 59.09% (52/88) | NR | NR | NR | 30 → 60 → 100 → 200 → 300 → 400 ng/kg/day (each 24 h) → 500 ng/kg/day |
| 9 | Boyiadzis M., 2023 | JNJ-63709178 | CD3-CD123 | Janssen | AML | 16.13% (10/62) | 3.23% (2/62) | NR | NR | NR | NR | Overall dose range explored: 0.15 – 4.8 μg/kg |
| 10 | Ravandi F., 2024 | AMG 330 | CD3-CD33 | Amgen | AML | 19.48% (15/77) | NR | NR | NR | NR | NR | Up to 6 dose steps before target dose |
| 11 | Hutchings M., 2025 | RO7283420 | CD3-HLA-A2-WT1 | Hoffmann-La Roche | AML | 34.21% (13/38) | 1.61% (1/62) | NR | 1.61% (1/62) | NR | NR | 1→3→12 mg Q3W IV (double step-up) |
| **Lymphoma** | | | | | | | | | | | | |
| 12 | Rothe A., 2015 | AFM13 | CD16A-CD30 | Affimed | HL | 3.57% (1/28) | NR | NR | NR | 3.57% (1/28) | NR | 0.01 → 0.04 → 0.15 → 0.5 → 1.5 → 4.5 → 7.0 mg/kg; weekly × 4 doses/cycle |
| 13 | Goebeler ME., 2016 | Blinatumomab | CD3-CD19 | Amgen | NHL | 22.37% (17/76) | 2.63% (2/76) | NR | NR | 1.32% (1/76) | 22.37% (17/76) | 5→15→60 µg/m²/day |
| 14 | Hutchings M,, 2021 | Glofitamab | CD3-CD20 | Hoffmann-La Roche | B- NHL | 2.92% (5/171) | 1.17% (2/171) | NR | 5.26% (9/171) | 0.58% (1/171) | NR | 2.5 →10 → 30 mg |
| 15 | Hutchings M,, 2021 | Epcoritamab | CD3-CD20 | Genmab | B- NHL | 0.00% (0/68) | 0.00% (0/68) | NR | NR | NR | NR | Cycle 1 (introduced at the 1.5 mg full-dose level onward) → full dose (0.0128–60 mg). |
| 16 | Bannerji R., 2022 | Odronextamab | CD3-CD20 | Regeneron | B-cell NHL | 8.28% (12/145) | 2.76% (4/145) | 4.83% (7/145) | 2.76% (4/145) | 2.07% (3/145) | NR | 3-step schedule in cycle 1 (days 1, 2, 8, 9, 15, 16), followed by full target doses weekly in cycles 2–4, then every 2 weeks |
| 17 | Wang L., 2026 | PSB202 | CD20-CD37 | Noncommunicable Chronic Diseases-National Science and Technology Major Project | B-NHL | 0.00% (0/15) | 0.00% (0/15) | NR | 0.00% (0/15) | NR | NR | 36 mg priming dose added 7 days prior to target dose after DLT at 100 mg |
| 18 | Thieblemont C., 2022 | Epcoritamab | CD3-CD20 | Genmab | Large B- NHL | 7.64% (12/157) | 0.64% (1/157) | 48.72% (38/78) | 5.73% (9/157) | 0.64% (1/157) | NR | C1D1: 0.16 mg → C1D8: 0.8 mg → C1D15: 15 mg |
| 19 | Viardot A., 2016 | Blinatumomab | CD3-CD19 | Amgen | DLBCL | 21.74% (5/23) | 0.00% (0/25) | NR | NR | NR | NR | 9 → 28 → 112 µg/day (weekly increases) |
| 20 | Katz DA., 2022 | Blinatumomab | CD3-CD19 | Amgen | DLBCL* | 7.14% (2/28) | 0.00% (0/28) | NR | NR | 3.57% (1/28) | NR | Cycle 1: 9 µg/day ×7d → 28 µg/day ×7d → 112 µg/day ×42d; Cycle 2: 9→28→112 µg/day with shorter final step (×14d). |
| 21 | Abramson JS., 2024 | Glofitamab | CD3-CD20 | Hoffmann-La Roche | DLBCL | 57.92% (106/183) | 2.78% (5/180) | NR | 2.22% (4/180) | NR | NR | Day 8: 2.5 mg → Day 15: 10 mg → Cycles 2–12: 30 mg (fixed) |
| 22 | Kim WS., 2025 | Odronextamab | CD3-CD20 | Regeneron | DLBCL | 13.39% (17/127) | 3.94% (5/127) | 46.67% (28/60) | NR | 0.79% (1/127) | NR | Two regimens used: 1→20 mg (n=67) and 0.7→4→20 mg (n=60); full dose 160 mg weekly thereafter |
| 23 | Budde LE., 2022 | Mosunetuzu-mab | CD3-CD20 | Genentech | FL | 4.44% (4/90) | 0.00% (0/90) | 42.50% (17/40) | 5.56% (5/90) | NR | NR | C1D1: 1 mg → C1D8: 2 mg → C1D15: 60 mg → C2D1: 60 mg → C3+: 30 mg q21d |
| 24 | Linton KM., 2024 | Epcoritamab | CD3-CD20 | Genmab | FL | 18.75% (24/128) | 0.00% (0/128) | 37.5% (48/128) | 6.25% (8/128) | 1.56% (2/128) | NR | 2-stage: 0.16 mg (day 1) → 0.80 mg (day 8) → 48 mg full doses; prophylaxis with prednisolone 100 mg |
| 25 | Falchi L., 2026 | Epcoritamab | CD3-CD20 | AbbVie and Genmab. | FL | 18.93% (46/243) | 1.65% (4/243) | 25.71% (9/35) | NR | 18.11% (44/243) | NR | 0.16 mg on C1D1 → 0.8 mg on C1D8 or a 0.16 mg on C1D1 → 0.8 mg on C1D8 → 3 mg on C1D15— before escalating to the full dose of 48 mg. |
| 26 | Kim WS., 2025 | Acimtamig | CD16A-CD30 | Affimed | PTCL | 12.04% (13/108) | 0.00% (0/108) | 81.48% (88/108) | NR | NR | NR | Flat dose 200 mg IV weekly |
| **Multiple myeloma** | | | | | | | | | | | | |
| 27 | Topp MS., 2020 | Pacanalotamab | CD3-BCMA | Boehringer Ingelheim | MM | 16.67% (7/42) | 0.00% (0/42) | 2.38% (1/42) | 0.00% (0/42) | 4.76% (2/42) | NR | Single-patient cohorts: 0.2, 0.4, 0.8, 1.6 µg/d. No step-up dose. |
| 28.1 | Chari A.., 2022 | Talquetamab (SQ) | CD3-GPRC5D | Janssen | MM | 2.31% (3/130) | NR | 56.15% (73/130) | NR | 3.08% (4/130) | NR | 405 µg/kg weekly (with 10 and 60 µg/kg step-up doses) and 800 µg/kg every other week (with 10, 60, and 300 µg/kg step-up doses). |
| 28.2 | Chari A.., 2022 | Talquetamab (IV) | CD3-GPRC5D | Janssen | MM | 3.92% (4/102) | NR | 48.04% (49/102) | NR | 1.96% (2/102) | NR | 0.5 to 180 µg/kg weekly or every other week, with the most active doses being 20, 60, and 180 µg/kg weekly. |
| 29 | Moreau P., 2022 | Teclistamab | CD3-BCMA | Janssen | MM | 1.21% (2/165) | 3.03% (5/165) | 48.48% (80/165) | 3.03% (5/165) | NR | 1.82% (3/165) | 0.06 → 0.3 → 1.5 mg/kg SQ weekly |
| 30 | Raab MS., 2023 | WVT078 | CD3-BCMA | Novartis | MM | 3.03% (1/33) | 0.00% (0/33) | 27.27% (9/33) | NR | 3.03% (1/33) | NR | No step-up dose: patients received intravenous WVT078 once weekly at the following dose levels: 3, 6, 12, 24, 48, 64, 96, 192, and 250 µg/kg |
| 22 | Plesner T., 2023 | RO7297089 | CD16a-BCMA | Genentech | MM | 0.00% (0/27) | 0.00% (0/27) | NR | NR | NR | NR | Not implemented; split first dose used in Arms B for 1080 mg and 1850 mg cohorts |
| 31 | Lesokhin AM., 2023 | Elranatamab | CD3-BCMA | Pfizer | MM | 13.82% (17/123) | 3.25% (4/123) | 30.08% (37/123) | 3.36% (4/119) | 15.45% (19/123) | NR | 12 mg (Day 1) → 32 mg (Day 4) → 76 mg QW; 98.8% of CRS events occurred within first 3 doses; 90.6% limited to step-up doses |
| 33 | Bumma N., 2024 | Linvoseltamab | CD3-BCMA | Regeneron | MM | 7.69% (9/117) | 2.56% (3/117) | 29.91% (35/117) | 7.69% (9/117) | 10.26% (12/117) | NR | 5 mg (day 1) → 25 mg (day 8) → full dose (day 15) |
| 34 | Rodriguez C., 2025 | Pacanalotamab | CD3-BCMA | Amgen | MM | 4.35% (1/23) | 0.00% (0/23) | 26.09% (6/23) | NR | NR | NR | Cohort 1 received a dose of 400 μg/day. In cohort 2, patients received 200 μg/day for the first 3 days in cycle 1 and 600 μg/day cycle 1 day 4 onwards (step-dosing); 600 μg/day in subsequent cycles. |
| 35 | Chari A., 2025 | Talquetamab | CD3-GPRC5D | Janssen | MM | 9.09% (14/154) | 0.00% (0/154) | 70.78% (109/154) | 10.17% (12/118) | 5.84% (9/154) | NR | 3 step-up doses: 0.01 mg/kg, 0.06 mg/kg, 0.3 mg/kg |
| 36 | Touzeau C., 2026 | Teclistamab | CD3-BCMA | Janssen | MM | 10.7% (NR/NR) | 6.53% (19/291) | NR | NR | 14.4% (NR/NR) | 69.10% (NR/NR) | 0.06 mg/kg → 0.3 mg/kg → 1.5 mg/kg weekly |

*Newly diagnosed DLBCL. Abbreviations: BsAb: Bispecific antibody; BCMA: B-cell maturation antigen; GPRC5D: G protein-coupled receptor family C group 5 member D; HLA-A2: Human leukocyte antigen A2; WT1: Wilms tumor 1; R/R: Relapsed/Refractory; BCP-ALL: B-cell precursor acute lymphoblastic leukemia; Ph-ve: Philadelphia chromosome-negative; Ph+ ALL: Philadelphia chromosome-positive acute lymphoblastic leukemia; AML: Acute myeloid leukemia; HL: Hodgkin lymphoma; NHL: Non-Hodgkin lymphoma; B-NHL: B-cell non-Hodgkin lymphoma; DLBCL: Diffuse large B-cell lymphoma; FL: Follicular lymphoma; PTCL: Peripheral T-cell lymphoma; MM: Multiple myeloma; SQ: Subcutaneous; IV: Intravenous; CRS: Cytokine release syndrome; ICANS: Immune effector cell-associated neurotoxicity syndrome; AEs: Adverse events; DLT: Dose-limiting toxicity; RP2D: Recommended phase II dose; QD: Once daily; TIW: Three times weekly; QW: Once weekly; Q3W: Every 3 weeks; q21d: Every 21 days; wk: Week; d: Day; C: Cycle; C1D1: Cycle 1 Day 1; C1D8: Cycle 1 Day 8; C1D15: Cycle 1 Day 15; NR: Not reported.
